# Supplementary material for: Comprehensive Sequence Analysis of Parvalbumins in Fish and Their Comparison with Parvalbumins in Tetrapod Species
Source: Biology (Basel). 2022 Nov 25;11(12):1713. doi: 10.3390/biology11121713 (PMC9774829; doi:10.3390/biology11121713)
Supplement: Supplementary file 1 [file biology-11-01713-s001.zip › Supplementary file S1.pdf]

Supplementary File S1

Sequences

This file contains the sequences used in the present study for comparison with indications of their database accessions. Some predictions of coding exon use were modified compared to database predictions to better fit with parvalbumin consensus sequences. The first part of this list, from page 2, includes "Species for which all parvalbumin genes were analyzed" in the order (as in Fig. 2 and Supplementary file S3) *Callorhinchus milii* (elephant shark; page 2), *Latimeria chalumnae* (coelacanth; page 2), *Xenopus tropicalis* (tropical clawed frog; page 3), *Gallus gallus* (chicken; page 4), *Ornithorhynchus anatinus* (platypus; page 4), *Sarcophilus harrisii* (Tasmanian devil; page 5), *Talpa occidentalis* (Iberian mole; page 5), *Mus musculus* (mouse; page 5), *Homo sapiens* (human; page 5), *Polypterus senegalus* (gray bichir; page 6), *Lepisosteus oculatus* (spotted gar; page 7), *Anguilla anguilla* (European eel; page 8), *Paramormyrops kingsleyae* (elephant fish; page 9), *Danio rerio* (zebrafish; page 10), *Onychostoma macrolepis* (page 11), *Clupea harengus* (Atlantic herring; page 12), *Ictalurus punctatus* (channel catfish; page 13), *Esox lucius* (Northern pike; page 14), *Gadus morhua* (Atlantic cod; page 15), *Takifugu rubripes* (fugu; page 16), *Seriola lalandi* (yellowtail amberjack; page 17), *Seriola dumerili* (greater amberjack; page 18), *Scophthalmus maximus* (turbot; page 19), *Sparus aurata* (gilthead seabream; page 20), *Oreochromis niloticus* (Nile tilapia; page 21), and *Oryzias latipes* (Japanese medaka; page 21).
This is followed by, from page 23, by "Other parvalbumin sequences" which include sequences in the alphabetic order of their Latin names from *Amphiuma means* (two-toed amphiuma; page 23), *Coryphaenoides rupestris* (roundnose grenadier; page 23), *Ctenopharyngodon Idella* (grass carp; page 23), *Cyprinus carpio* (common carp; page 23), *Lates calcarifer* (barramundi perch; page 24), *Mustelus griseus* (spotless smooth-hound shark; page 24), *Pelophylax lessonae* (pool frog; page 24), *Plecoglossus altivelis* (ayu; page 25), *Salmo salar* (Atlantic salmon; page 25), *Sardinops melanostictus* (crimson seabream; page 25), *Scomber japonicus* (Pacific mackerel; page 25), and *Triakis semifasciata* (leopard shark; page 25).

Species for which all parvalbumin genes were analyzed

(The order of species listing is as in main text Fig. 2 and Supplementary file S3)

***Callorhinchus milii* (elephant shark)_**

>Callorhinchus_milii_(elephant_shark)_6258
The name is derived from Ensembl dataset Callorhinchus_milii-6.1.3 gene ENSCMIG00000006258.
Sequence: GenBank XP_007891758
MLVTDILAASDISNAIKECQALESFDFKKFFQTSGLSRKSADEIKNVFLAMDDDDSGYIELDELKFFLQRFSP
IARVLTEAETEAFLKAADTDGDERIGTQEFVTLVMS

>Callorhinchus_milii_(elephant_shark)_6256
The name is derived from Ensembl dataset Callorhinchus_milii-6.1.3 gene ENSCMIG00000006256.
Sequence: GenBank NP_001279781
MAITDILAASDIDSAISQFSAPDSLNLKKFFEASGLSRKSADEVAKVFEIMDQDNNGFIEKEEIKKFLQFFSS
RARTLTEAEIQIFIAAGDKDSDGKIGITEFQRMVSA

>Callorhinchus_milii_(elephant_shark)_6255
The name is derived from Ensembl dataset Callorhinchus_milii-6.1.3 gene ENSCMIG00000006255.
Sequence: GenBank XP_007891755
MSITDILTAADIASAVSQCSAVNSFCHKKFFTTCGLSKKSETDVKKVFKVLDQDQSGYIEDAELKLFLQSFST
GARELTEAETAAFLKAGDEDGDGMIGADEFWKLVSA

>Callorhinchus_milii_(elephant_shark)_1941
The name is derived from Ensembl dataset Callorhinchus_milii-6.1.3 gene ENSCMIG00000011941.
Sequence: GenBank XP_007907985
MGITDYLDAGDVAAALAQCKAADSFSFKQFCATSGLAKKSSDTVNKIFNIIDEDGSGYIEAKELKFFLQNFSS
DARELGDSEIKTVLSAMGMSDDGKMNATEFQKLVSA

>Callorhinchus_milii_(elephant_shark)_pvalb-alpha
Sequence: GenBank AFP11872
MSINKILSAADITKALGEFKGADSFDHKKFFHMIGLKKKNAKDVEAVFYILDKDKSGFIEEDELKSVLKCFAP
EGRDLSEKETKDLLTAGDEDGDGKIGVSEFIQLVANS

***Latimeria chalumnae* (coelacanth)**

>Latimeria_chalumnae_(coelacanth)_oncomodulin
Sequence: GenBank XP_014345346
MSITDILSSSDIAAALKECQAPESFNCKKFFETSGLAKKSPTDVKNVFKILDNDQSGYIEEEELQFFLQRFDA
EARTLSMNETKKFMQAADGDGDGMIGAEEFQAMVHS

>Latimeria_chalumnae_(coelacanth)_XP_005998420
Sequence: GenBank XP_005998420
MALTDILAAADITSALKSCQAEGSFNHKTFFSQVGLTGKSKAQVSKVFGILDQDKSGFIEEEELKLFLQNFSS
SARALSDAETKAFLAAGDSDGDGKIGVDEFSSMVEA

>Latimeria_chalumnae_(coelacanth)_XP_005998419
Sequence: GenBank XP_005998419
MAFGGILKEADITAALKGCQAADSFNFKKFFVEVGLHGKSDKDAEAIFNILDQDGSGYIEEDELKLFLQNFSA
SARALTDAETKAFMAAGDVDGDGKIGVVEFQTLVKA

>Latimeria_chalumnae_(coelacanth)_XP_005998418
Sequence: GenBank XP_005998418
MAVAKLLAAADVTAALEGCKADDSFNHKVFFQKTGLAKKSNEELEAIFKILDQDKSGFIEDEELQLFLQNFSA
GARTLTKTETETFLKAGDSDGDGKIGVDEFQKLVKA

>Latimeria_chalumnae_(coelacanth)_pvalb-alpha
Sequence: GenBank XP_006012166
MTKKMSEILKAEDIDKALNTFKEAGSFDHHKFFNLVGLKGKPDDTLKEVFGILDQDKSGYIEEEELKFVLKGF
GAAGRELTANETKALLKAGDQDGDDKIGVDEFTNLVKAA

Xenopus tropicalis **(tropical clawed frog)**

(The "OCM3" and multiple "OCM4" genes are no oncomodulin genes, but with these names we follow the database annotations)

> Xenopus_tropicalis_(tropical_clawed_frog)_OCM1
Sequence: GenBank XP_002931997
MSITDVLSAADIAAALRECQAPDTFNYKKFFKTCGLTKKSPNEVKQVFGVMDNDESGYVEEDELKYILQRFDP
SARVLTSSEAKDFMAGVDHDGDGKIGVEEFQAVVHTCASQ

> Xenopus_tropicalis_(tropical_clawed_frog)_OCM4.6
Sequence: GenBank NP_001123669
MAFGGLLSEADISAALQECQAADSFNYKSFFARSGLSKKSADDVKNVFAILDQDRSGFIEEDELKLFLQNFSA
SARALTDAETKAFLGAGDSDGDGKIGVEEFQSLVKQ

> Xenopus_tropicalis_(tropical_clawed_frog)_OCM4.5
Sequence: GenBank NP_001120120
MAFGGILSEADISAALQNCQAPDSFNFKTFFAQSGLSSRSADDVKNVFGILDQDRSGFIEEDELKLFLQNFSA
GARALTDAETKAFLAAGDSDGDGKIGVEEFQALVRP

> Xenopus_tropicalis_(tropical_clawed_frog)_OCM4.4
Sequence: GenBank NP_001015897
MALGALLSQADISAALQACQAPDSFNYKSFFAQSGLSSKSVDDVRNAFAILDQDKSGFIEEDELKLFLQNFSA
GARALTDAETKAFLNAGDSDGDGKIGVDEFQALVKA

> Xenopus_tropicalis_(tropical_clawed_frog)_OCM4.3
Sequence: GenBank NP_001107673
MAFGGILSDADISSALKNCQAPDSFNFKSFFAQSGLSSKSDNDVRNVFAIIDQDKSGFIEEDELKLFLQNFSA
GARALTDAETKAFLNAGDSDGDGKIGVDEFQSLVKN

> Xenopus_tropicalis_(tropical_clawed_frog)_OCM4.2
Sequence: GenBank NP_001016487
MAFGGILSDADVSAALQNCQAPESFNFKTFFAQSGLSSKSDNDVRNVFAILDQDRSGFIEEDELKLFLQNFNA
GARALTDAETKAFLNAGDSDGDGKIGVDEFQALVKN

> Xenopus_tropicalis_(tropical_clawed_frog)_OCM4.1
Sequence: GenBank NP_001011134

MAFGGILSEADVSAALQSVQAADSFNYKSFFAKSGLSSKSADDVKKVFAILDQDKSGFIEEDELKLFLQNFNS
GARALTDAETKAFLAAGDSDGDGKIGVDEFQALVKA

> Xenopus_tropicalis_(tropical_clawed_frog)_OCM3
Sequence: GenBank NP_001025657
MSITDILSAKDIDAALSSVSAAESFQHKSFFDKCGLTGKSNDQVRKVFEILDRDKSGFIEEDELQLFLQNFKS
NARALSAAETKAFLKAGDSDGDGKIGVDEFQALVKA

> Xenopus_tropicalis_(tropical_clawed_frog)_pvalb-alpha
Sequence: GenBank XP_002934266
MSMTDVLAAADIKNALGAFGAPDSFNHKKFFELCGLKKKSPDDVKKVFHILDKDRSGFIEEDELKLLLQGFAT
DGRALSDKETKALLAAGDKDGDGKIGVDEFTALVAES

Gallus gallus (chicken)

>Gallus_gallus_(chicken)_oncomodulin_(CPV3)
Sequence: GenBank NP_989753
MSLTDILSPSDIAAALRDCQAPDSFSPKKFFQISGMSKKSSSQLKEIFRILDNDQSGFIE
EDELKYFLQRFECGARVLTASETKTFLAAADHDGDGKIGAEEFQEMVQS

>Gallus_gallus_(chicken)_1286
The name is derived from Ensembl dataset GRCg6a gene ENSGALG00000021286.
Sequence: Modification of GenBank XP_025011090
MAFAGILTEAEIAAGLQSCQAADSFNYKTFFVKVGLNSKSKDQLAKVFGILDQDRSGFIEEDELKLFLQNFSA
SARALTDAETKAFLAAGDSDGDGKIGVDEFQALVKS

>Gallus_gallus_(chicken)_ATH
Sequence: GenBank NP_001007478
MAITDILSAKDIESALSSCQAADSFNYKSFFSTVGLSSKTPDQIKKVFGILDQDKSGFIEEEELQLFLKNFSS
SARVLTSAETKAFLAAGDTDGDGKIGVEEFQSLVKA

>Gallus_gallus_(chicken)_pvalb-alpha
Sequence: GenBank NP_001152787
MAMTDVLSAEDIKKAVGAFSAAESFNYKKFFEMVGLKKKSPEDVKKVFHILDKDRSGFIEEEELKFVLKGFTP
DGRDLSDKETKALLAAGDKDGDGKIGADEFATMVAES

Ornithorhynchus anatinus (platypus)

>Ornithorhynchus_anatinus_(platypus)_oncomodulin
Sequence: GenBank XP_039770783
MSITDVLSAADIAAALKECQDPDSFNHKQFFQTSGLAKKSASQVKDIFRFIDNDQSGFLDEDELKFFLQKFES
GARVLTSSETKSLMAAADHDGDGKIGADEFQEMVHS

>Ornithorhynchus_anatinus_(platypus)_XP_001512095
Sequence: GenBank XP_001512095
MAMTDILSAKDIEAALSSCKAADSFNYKAFFSTVGLSKKTPDQVKKVFGILDQDKSGFIEEDELQLFLQNFSS
SARVLTAAETKAFLTAGDTDGDGKIGVDEFQNLVKS

>Ornithorhynchus_anatinus_(platypus)_pvalb-alpha
Sequence: GenBank XP_028935194
MAMTDLLSADDIKKAVGAFSAAESFNHKKFFELVGLKKKSKDEVQKVFHILDKDKSGFIEEDELKFVLKGFIE
DGRDLSDKETKTLLAAGDKDGDGKIGVEEFTTLVAES

***Sarcophilus harrisii* (Tasmanian devil)**

>Sarcophilus_harrisii_(Tasmanian_devil)_oncomodulin
Sequence: GenBank XP_031799913
MSITDVLSAADIAAALEECQDPDSFNHKKFFETSGLSKMSASQVKDIFRFIDNDQSGYLDEEELKFFLQKFES
GARVLTSSETKSLLEAADNDGDGKIGADEFQEMVHS

>Sarcophilus harrisii (Tasmanian devil)_XP_003761513
Sequence: GenBank XP_003761513
MSMTDILSPKDIEAALSSCKAAGSFDYKIFFSKVGLSGKTPEQVKKVFSILDQDKSGFLEEDELQNFLQNFAP
GARTLTPDETKIFLTAGDSDGDGKIGVDEFQSMAKS

>Sarcophilus harrisii (Tasmanian devil)_pvalb-alpha
Sequence: GenBank XP_003771180
MSITDLLSVEDIKKAVAAFAAAESFNHKKFFEMVGLKKKNKQDVEKVFHILDKDKSGFIEEDELKFILKGFTP
DGRDLSDKETKALLAAGDKDGDGKIGAEEFSALVAES

***Talpa occidentalis* (Iberian mole)**

>Talpa_occidentalis_(Iberian_mole)_oncomodulin
Sequence: GenBank XP_037360420
MSITDLLSADDIAAALQECQDPDTFEPQKFFQTSGLSKMSASQVKDVFRFIDNDQSGYLDEEELKFFLQKFES
GARELTESETKSLMAAADNDGDGKIGADEFQEMVHS

>Talpa_occidentalis_(Iberian_mole)_XP_037360422
Sequence: GenBank XP_037360422
MAMTDIVSLKDIEAALSSCKDPGSFNHKAFFSEVGLSSKDIDLVRKAFAILDQDKSGFIEEEELKLFLKNFSP
SARVLTDKETKAFLEAGDSDGDGKIGVDEFQSLVKA

>Talpa_occidentalis_(Iberian_mole)_pvalb-alpha
Sequence: GenBank XP_037369536
MSMTDLLSAEDIKKALGAFAAVDSFDHKKFFQMVGLKKKSADDVKKVFHILDKDKSGFIEEDELGSILKGFSA
DARDLSARETKALLAAGDKDGDGKIGVEEFSALVAES

***Mus musculus* (mouse)**

>Mus_musculus_(mouse)_oncomodulin
Sequence: GenBank NP_149028
MSITDILSADDIAAALQECQDPDTFEPQKFFQTSGLSKMSASQLKDIFQFIDNDQSGYLDEDELKYFLQRFQS
DARELTESETKSLMDAADNDGDGKIGADEFQEMVHS

>Mus_musculus_(mouse)_pvalb-alpha
Sequence: GenBank NP_001317615
MSMTDVLSAEDIKKAIGAFAAADSFDHKKFFQMVGLKKKNPDEVKKVFHILDKDKSGFIEEDELGSILKGFSS
DARDLSAKETKTLLAAGDKDGDGKIGVEEFSTLVAES

***Homo sapiens* (human)**

>Homo_sapiens_(human)_OCM1
Sequence: GenBank NP_001091091

MSITDVLSADDIAAALQECRDPDTFEPQKFFQTSGLSKMSANQVKDVFRFIDNDQSGYLDEEELKFFLQKFES
GARELTESETKSLMAAADNDGDGKIGAEEFQEMVHS

>Homo_sapiens_(human)_OCM2
Sequence: GenBank NP_006179
MSITDVLSADDIAAALQECQDPDTFEPQKFFQTSGLSKMSASQVKDVFRFIDNDQSGYLDEEELKFFLQKFES
GARELTESETKSLMAAADNDGDGKIGAEEFQEMVHS

>Homo_sapiens_(human)_pvalb-alpha
Sequence: GenBank NP_001302461
MSMTDLLNAEDIKKAVGAFSATDSFDHKKFFQMVGLKKKSADDVKKVFHMLDKDKSGFIEEDELGFILKGFSP
DARDLSAKETKMLMAAGDKDGDGKIGVDEFSTLVAES

>Homo_sapiens_(human)_calmodulin-1
Sequence: GenBank NP_001350599
MQADQLTEEQIAEFKEAFSLFDKDGDGTITTKELGTVMRSLGQNPTEAELQDMINEVDADGNGTIDFPEF
LTMMARKMKDTDSEEEIREAFRVFDKDGNGYISAAELRHVMTNLGEKLTDEEVDEMIREADIDGDGQVNY
EEFVQMMTAK

Polypterus senegalus **(Gray bichir)**

>Polypterus_senegalus_(gray_bichir)_3080
The name is derived from NCBI gene annotation LOC120543080.
Sequence: GenBank XP_039631865
MSLTSILCPDDIANALKDCQAENSFNHKKFFQLCGMTKKSPKEIKEAFAILDNDRSGFIEEEELKFFLQRFSP
GARVLTEAETKSFLAAADDDNDGKIGAAEFQAMVLS

>Polypterus_senegalus_(gray_bichir)_2990
The name is derived from NCBI gene annotation LOC120542990. Sequence:
GenBank XP_039631723
MAFSGLLKDEDIKAALEACKAPDSFNYKNFFAKVGLTGRSADDVKKAFAVLDQDKSGFIEEDELKLFLQNFCS
GARALTDAETKAFLAAGDSDGDGKIGVDEFASLVKA

>Polypterus_senegalus_(gray_bichir)_2675
The name is derived from NCBI gene annotation LOC120542675. Sequence:
GenBank XP_039631215
MSFAGVLKDDDIKAALQACQAPDSFNYKTFFAKVGLTGRSAEDVKKAFAVLDQDKSGFIEEDELKLFLQNFQS
GARALTDKETKAFMTAGDSDGDGKIGVDEFAALVKA

>Polypterus_senegalus_(gray_bichir)_3078
The name is derived from NCBI gene annotation LOC120543078.
Sequence: GenBank XP_039631862
MAFAGLLKDEDIAAAIKNCQAADSFNHKTFFSQVGLANKSAADVAKVFKILDQDHSGFIEEDELKLFLQNFQS
SARALTDAETKTFLAAGDSDGDGKIGVEEFQALVK

>Polypterus_senegalus_(gray_bichir)_2892
The name is derived from NCBI gene annotation LOC120542892. Sequence:
GenBank XP_039631543
MTMTDVLSAADIAAALNACQDVGSFNYKSFFATVGLSRKSPADVQRVFDIIDQDRSGFIEEEELQLFLQNFSK
NARVLTVAETRAFLKAGDLDNDGKLGAEEFTALVHT

>Polypterus_senegalus_(gray_bichir)_6869

The name is derived from NCBI gene annotation LOC120536869. Sequence: GenBank XP_039621339
MAFAGILKDDDIKAAVQAFQAPDSFDHKAFFSKVGMTDKSPDDVKKIFAILDQDKSGFIEEAELKFFMQNFEA
GARVLTEKENKALLAAIDKDGDGKIGCEEFVALVKG

>Polypterus_senegalus_(gray_bichir)_pvalb-alpha
Sequence: GenBank XP_039621268
MLMTDLLKADDISKAVGAFKAAESFDHKKFFEMVGLKKKTPDEVKKVFQILDQDHSGFIEEEELKFVMKGFSK
DGRDLSDAETKKFMKAGDKDGDGKIGIDEFATLVAES

>Polypterus_senegalus_(gray_bichir)_6197
The name is derived from NCBI gene annotation LOC120536197. Sequence: GenBank XP_039620468
MAFAGILEKEDIETAIETCQAPDTFDHKAFFAQVGLTTKPAEELKKIFAVLDQDKSGYIEETELKFFLQNFQA
GARVLTDKENKTLLTAGDADADGKMGPDEFVALVKS

Lepisosteus oculatus (spotted gar)

>Lepisosteus_oculatus_(spotted_gar)_ 8552
The name is derived from Ensembl dataset LepOcu1 gene ENSCMIG00000006258. Sequence: Encoded by Ensembl dataset LepOcu1 transcript pvalb8-201
MSLNSILSSDAIEAALKDCQAPDSFNQKKFFQLCGLTKKSPQEVKDVFRILDNDASGFIEEEELKFFLQRFSP
SARVLTAAETKNFLSAADDDNDGKIGCEEFQAMVLS

>Lepisosteus_oculatus_(spotted_gar)_ 8560
The name is derived from Ensembl dataset LepOcu1 gene ENSLOCG00000008560. Sequence: Encoded by Ensembl dataset LepOcu1 transcript ENSLOCT00000010427
MAFAGILKDEDIAAALQACQAADSFNYKTFFAKVGLAGRSADDVKKAFAVLDQDKSGFIEEDELKLFLQNFKA
DARVLTDAETKAFLAAGDSDGDGKIGVDEFAALVKA

>Lepisosteus_oculatus_(spotted_gar)_ 8573
The name is derived from Ensembl dataset LepOcu1 gene ENSLOCG00000008573. Sequence: Encoded by modified Ensembl dataset LepOcu1 transcript ENSLOCT00000010439 (there is no apparent exon-4 sequence, and also because the exon-3 encoded sequence is unusually short, this may be a pseudogene; however, the quality of the avialabl genomic sequence information for the C-terminal part of the protein is not sufficient to make firm conclusions)
MAFAGILKDEDVAAALQACQAADSFNYKTFFAKVGLAGRSAEDVKKAFAVLDQDKSGFIEEDELKLFLQNFKA
DARVLTDAETKAFLAAGDS

>Lepisosteus_oculatus_(spotted_gar)_8585
The name is derived from Ensembl dataset LepOcu1 gene ENSLOCG00000008585. Sequence: Encoded by Ensembl dataset LepOcu1 transcript ENSLOCT00000010450
MAFAGILKDADIAAALKTCEAAGSFNHKTFFAEVGLASKPDSEAEKVFKILDQDQSGFIEEDELKLFLQNFSA
SARVLTDAETKTFLAAGDSDGDGKIGVDEFRALTK

>Lepisosteus_oculatus_(spotted_gar)_8594
The name is derived from Ensembl dataset LepOcu1 gene ENSLOCG00000008594.

Sequence: Encoded by modified Ensembl dataset LepOcu1 transcript ENSLOCT00000010463
MAFSGLLREEDITAALQACQAAGSFNHKAFFAQVGLASDSAVSAEKVFRILDRDQSGFIEEEELKLFLQNFKA
DARVLTDAETKAFLAAADLDSDGKIGAQEFRSLMK

>Lepisosteus_oculatus_(spotted_gar)_8606
The name is derived from Ensembl dataset LepOcu1 gene ENSLOCG00000008606.
Sequence: Encoded by Ensembl dataset LepOcu1 transcript ENSLOCT00000010476
(there is no exon-4, according to the Genbank transcript accession GFIM01033536, and such exon is also not predicted in Ensembl)
MALSGLLAAGAVAAALTACQATGSFDHKAFFAAVGLSKKSPADVEKVFKVLDQNNSGFIEEDELQLFLQSFSK
GARVLTAAETRAFLLAGDSDGDGKIGVEGT

>Lepisosteus_oculatus_(spotted_gar)_pvalb-alpha
Sequence: GenBank GFIM01023648 (TSA transcript information).
(There is incomplete genomic information on GenBank NW_006271359 and Ensembl dataset LepOcu1 scaffolds AHAT01044600 and AHAT01044594)
MSMTDILKAEDIKAAMDAFKAAESFDHKKFFKMVGLKAKSAEDVKKAFKVLDVDQSGFIEEDELKFVLKGFSK
DGRDLSDKETKAFLVAGDKDGDGKIGIDEFAALVAEA

Anguilla anguilla (European eel)

>Anguilla_anguilla_(European_eel)_pvalb1
Sequence: GenBank XP_035253170
MAFAGVLKDADITAALAACQGADSFDYKSFFAKVGLSGKTPDEIKKAFSIIDQDNSGFIEEDELKLFLQNFSA
GARALTDKETKTFLAAGDTDGDGKIGIDEFAALVKA

>Anguilla_anguilla_(European_eel)_pvalb3
Sequence: GenBank XP_035258683
MAFAGVLNDADITAALQACQAADSFNYKSFFAKVGISGKSADIVKKVFAILDQDKSGFIEEDELKLFLQNFSG
SARALTDAETSAFLKAGDTDGDGKIGIDEFSALVKA

>Anguilla_anguilla_(European_eel)_pvalb4
Sequence: GenBank XP_035253204
MAFAGVLKDADITAALEACKAADSFNYKAFFAKVGLATKSADDIKKAFNIIDQDKSGFIEEDELKLFLQNFSK
TARALTDKETKAFLHAGDTDGDGKIGIDEFAAVVKA

>Anguilla_anguilla_(European_eel)_pvalb5
Sequence: GenBank XP_035253199
MSFSGILKDEDIKAAVLACEAPGTFDYKLFFTQVGLLSQQDVEGKTVFNVLDRDQSGFIEEAELKFFLQNFCL
GARELTEAETKAFISAVDRDGDGKIGVEEFCTLLK

>Anguilla_anguilla_(European_eel)_pvalb6
Sequence: GenBank XP_035253026
MAMKDILKAEDIKKAMDACKVADTFDHKKFFEMVGLKTRSTDDLKKAFLALDVDNSGFIEEEELKFFLKGFAT
DGRDLTDKETKAFLRAADKDGDGKIGMEEFAAMVRE

>Anguilla_anguilla_(European_eel)_pvalb7
Sequence: GenBank XP_035261400

MAMKDILNADEIKKALDAFKAAESFNHMKFFEMIGLKAKSAEDVKKVFKVLDVDNSGFIEEEELKFVLKGFAS
DGRDLTDKETKAFLEAADRDGDGKIGVDEFEALVHE

>Anguilla_anguilla_(European_eel)_pvalb8
Sequence: GenBank XP_035253329
MSLTSILSAGAIDSAIKDCSAPDSFCSKKFFQICGLTKKSPQEVKEVFRVLDNDDSGYIEEEELKFFLQRFSS
GARVLTEKETKAFLVAADGDSDGKIGVQEFQKLVLS

>Anguilla_anguilla_(European_eel)_ pvalb9
Sequence: GenBank XP_035258685
MSLTSILAADAIENAIKDCQAPDSFNSKKFFQLSGLTKKSPQEVKDVFCILDNDGSGFIEEEELKFFLQRFSP
GARLLTDKETKSFLSDADDDSDGKIGAEEFQAMVLS

Anguilla_anguilla_(European_eel)_pvalb10
Sequence: GenBank XP_035259276
MAMTDLLTMSDITSAITACQDADSFNLKAFFRDVGLSKKSPLDIEKVFKILDQDKSGFIEHDELQLFLQNFSK
GARSLTAAETKAFLQAGDMDGDGKIGLEEFSALVKAS

>Anguilla_anguilla_(European_eel)_pvalb-like
Sequence: GenBank XP_035261997
MADDFSLQVKKVTLAMGASLSDQDIDRIPREMRMQGNFNYSKFFEYMRQYKTSDQQEEVIKKAFQVLDKDGSG
YIEWNEIKYILSTVPSSVPIVPLSDEEAEAVIQAADADGDGRIDFREFSDLVKLEKKPRK

Paramormyrops kingsleyae **(Elephant fish)**

>Paramormyrops_kingsleyae_(elephant_fish)_pvalb1
Sequence: GenBank XP_023691426
MALASVLKEADITAAMKDCQAADSFNYKSFFAKVGLSSKSADDVKKAFAIIDQDNSGFIEEDELKLFLQNFSA
GARVLTDGETKTFLAAGDSDGDGKIGVDEFAALVKA

>Paramormyrops_kingsleyae_(elephant_fish)_pvalb2
Sequence: GenBank XP_023664883
MAFAGILKEEDVAAALKDCAAPDSFNHKSFFAKVGLASKSADDVKKAFAVIDQDKSGFIEEDELKLFLQNFSA
SARALTDAETKTFLAAGDSDGDGKIGADEFAAIVKA

>Paramormyrops_kingsleyae_(elephant_fish)_pvalb3
Sequence: GenBank XP_023664886
MAFAGILNDADITAALQACQAADSFSHKAFFEKVGLSGKSADDVKKAFAVIDQDKSGFIEEDELKLFLQNFSA
GARVLTDTETKTFLKAGDTDGDGKIGIDEFTALVKS

>Paramormyrops_kingsleyae_(elephant_fish)_pvalb4
Sequence: GenBank XP_023691425
MAFAGILNDGDITAALDACKAADSFSYKAFFAKVGLSSKSADDVKKAFAIIDQDKSGFIEEDELKLFLQNFSA
SARALTDAETKTFLKAGDSDGDGKIGADEFAALVKA

>Paramormyrops_kingsleyae_(elephant fish)_pvalb5(probably_pseudogene)
Sequence: Ensembl dataset PKINGS_01 gene pvalb5 (only the last two exons look normal and we could not find proper sequences for the first two exons, despite that the available genomic sequence information seems to be of good quality)
LFLQSFSSGARKLTEADTKDLLRTGDQDGDSKIGMEEFCSLMKRSEEV

>Paramormyrops_kingsleyae_(elephant fish)_pvalb6
Sequence: GenBank XP_023672368
MAMNDILKAEDIKKALDAFKAADTFDHKKFFEMVGLKNKSTDDVKKAFLALDVDNSGFIEEEELKFVLKGFST
DGRDLTDKETKAFLQAADKDGDGKIGMEEFAALVLE

>Paramormyrops_kingsleyae_(elephant fish)_pvalb7
Sequence: GenBank XP_023670307
MAMKDLLKAEDIKKALDAFKAADTFDPLKFFDMVGLKAKSSEDVKKAFKALDVDNSGFIEEEELKFVLKGFAS
DGRDLTDKETKAFLALADKDGDGKIGAEEFEALVHQ

>Paramormyrops_kingsleyae_(elephant_fish)_pvalb8
Sequence: GenBank XP_023691172
MSLTSVLSADAIANALKECKAPDTFCHKKFFQTCGLTKKTSQEVKNVFRILDDDGSEFIEEDELQFFLQRFSP
SARVLTESETKKFMLALDEDNDGKIGIDEFEHAVLS

>Paramormyrops_kingsleyae_(elephant_fish)_pvalb9
Sequence: GenBank XP_023665037
MSLTSILSADAIENAIKDCQAPDSFCYKKFFQLCGLSSKTPQEVKDVFRCIDDDDSGFIEDEELKFFLQKFSP
SARLLTEKETQAILNACDDDGDGKIGVDEFQVLVLS

Danio rerio **(Zebrafish)**

>Danio_rerio_(zebrafish)_pvalb1
Sequence: GenBank NP_991135
MAVGALLAAADVDAALAACQAADSFDYKSFFAKVGLSAKSADEVKKAFAIIDQDNSGFIEEEELKLFLQNFKA
NARVLTDKETKAFLSAGDSDGDGKIGAEEFAALVKA

>Danio_rerio_(zebrafish)-pvalb2
Sequence: GenBank NP_571591
MAFAGILKDEDVAAALKDCAAADSFNYKNFFAKVGLSAKSPDDIKKAFFVIDQDKSGFIEEDELKLFLQNFSA
GARALTDAETKAFLSAGDSDGDGKIGVDEFALLVKA

>Danio_rerio_(zebrafish)_pvalb3
Sequence: GenBank NP_956506
MAFAGILNEADITAALQACQAADSFDYKSFFAKVGLSAKTPDDIKKAFAVIDQDKSGFIEEDELKLFLQNFSA
GARALTDAETKAFLKAGDSDGDGKIGVDEFASLVKA

>Danio_rerio_(zebrafish)_pvalb4
Sequence: GenBank NP_997948
MAFAGVLNDADISAALEACKAADSFNHKSFFAKVGLASKSADEVKKAFAIIDQDKSGFIEEEELKLFLQNFKA
DARALTDGETKTFLKAGDSDGDGKIGIDEFAALVKA

>Danio_rerio_(zebrafish)_pvalb5
Sequence: GenBank NP_997050
MALAGILKQDDIAAAMQACQAQGSFNHESFFEQVGLIGRASSDGEKVFKALDQDKSGYIEKEELKRFLQNFCS
KARELTEAETNTLLAAGDKDGDGKIGIEEFCALLK

>Danio_rerio_(zebrafish)_pvalb6
Sequence: GenBank NP_991136
MAMSSILNHDDIKKALDACKAPDSFNHKSFFEMVGLKAKASDDVKKAFHLLDADNSGFIEEEELKFVLKAFAT
DGRDLTDKETKAFLQAADKDGDGKIGAEEFAALVRE

>Danio_rerio_(zebrafish)-pvalb7
Sequence: GenBank NP_991137
MAMKNLLKDDDIKKALDQFKAADSFDHKKFFDVVGLKALSADNVKLVFKALDVDASGFIEEEELKFVLKGFSA
DGRDLTDKETKAFLAAADKDGDGKIGIDEFEALVHE

Danio_rerio_(zebrafish)_pvalb8
Sequence: GenBank NP_891982
MSLTSILSADAIDCALKDCQAPDSFSPKKFFQLCGMTKKSPQDVKNIFNILDNDASGFIEEDELKFFLQRFSG
GARVLTDKETKAFLAAADDDSDGKIGAEEFQAMVLS

>Danio_rerio_(zebrafish)-pvalb9
Sequence: GenBank NP_891983
MSLTSILSAEAIENAVKDCQAPDSFCYKKFFQLCGLSQKTPQEVKDVFRIIDEDNSGFIEEAELKFFLQRFFP
GARTLTEKEIKSLLTAADDDSDGRIGVDEFQTMVSS

Onychostoma macrolepis (a diploid cyprinid fish without an English name)

>Onychostoma_macrolepis _pvalb1
Sequence: Modification of GenBank KAF4115537
MAVAAMLAAADVDAAIAACQAADSFNYKAFFAKVGLSAKSADDVKKAFAIIDQDNSGFIEEDELKLFLQNFKA
GARALTDKETKAFLTAGDSDGDGKIGAEEFAALVKA

>Onychostoma_macrolepis _pvalb2
Sequence: GenBank KAF4106695
MAFAGILKDEDVAAALKDCAAADSFNYKNFFAKVGLSAKTPDDIKKAFFVIDQDKSGFIEEDELKLFLQNFSA
GARALTDGETKAFLSAGDSDGDGKIGVDEFALLVKA

>Onychostoma_macrolepis _pvalb3
Sequence: GenBank KAF4106696
MAFAGILNDADITAALQACQAADSFNYKSFFAKVGLSAKTPDDIKKAFAVIDQDKSGFIEEDELKLFLQNFSA
GARALTDAETKAFLKAGDSDGDGKIGIDEFAVMVKA

>Onychostoma_macrolepis _pvalb4
Sequence: Modification of GenBank KAF4115536
MAFSGVLKDADITAALEACKAADSFNHKAFFAKVGLTSKSADDVKKAFAIIDQDKSGFIEEDELKLFLQNFKA
NARALTDAETKTFLKAGDSDGDGKIGVDEFAALVKS

>Onychostoma_macrolepis _pvalb5
Sequence: GenBank KAF4115538
MALAGHLKQDDIAAAIQACQAPGSFNYQTFFEHVGLAGKAASDGEKVFKALDQDKSGYIEKEELKRFLQNFCP
KARELTEAETNTLLAAGDSDGDGKIGMEEFCGLLK

>Onychostoma_macrolepis _pvalb6
Sequence: Modification of GenBank KAF4116378
MAMNSILNPDDIKKALDAFQAVDSFDPKRFFEMVGLKAKSAEDVKKAFHALDADNSGFIEEEELKFVFKHFAT
DGRDLSDKETKAFLQAADKDGDGKIGAEEFAALVRE

>Onychostoma_macrolepis _pvalb7

Sequence: GenBank KAF4097886
MAMKNLLKEDDIKKALDQFKAADSFDHKKFFDVVGLKALSAENVKLVFKALDVDASGFIEEEELKFVLKGFSA
DGRDLTDKETKAFLAAADKDGDGKIGIDEFEALVHE

>Onychostoma_macrolepis _pvalb8
Sequence: GenBank KAF4115535
MSLSSVLSADAIDSALKDCQAPDSFNPKKFFQLCGLTKKSPQEVKNVFNILDNDASGFIEEEELKFFLQRFSP
GARVLTDKETKGFLSAADDDSDGKIGADEFQAMVLS

>Onychostoma_macrolepis _pvalb9
Sequence: GenBank KAF4106697
MSLTSILSAEAIENAVKDCQAPDSFCYKKFFQLCGLSQKSPQEVKDVFRIIDEDNSGFIEEAELKFFLQRFFP
GARTLTEKEIKSLLTAADDDSDGRIGVDEFQTMVLS

>Onychostoma_macrolepis _pvalb10
Sequence: GenBank KAF4106694
MAVTDVLAASDISTAINACKAKDSFSPKTFFATVGLSKKSPPEIEKVFKMLDQDKSGFIEQDELQLFLQNFSK
GARALTAAETKAFLMAGDLDGDGKIGWEEFSALVNAS

***Clupea harengus* (Atlantic herring)**

>Clupea_harengus_(Atlantic_herring)_pvalb1
Sequence: Encoded by Ensembl dataset Ch_v2.0.2 transcript pvalb4-204
MAFAGLLSDADIAAALGACTAADTFDHKSFFKKVGLSGKSADDVKKAFYIIDQDKSGFIEEEELKLFLQNFKA
GARALSDKETKAFLAAGDADGDGMIGVDEFAVMVKA

>Clupea_harengus_(Atlantic_herring)_pvalb2A
Sequence: GenBank XP_012694367
MAFAGILKDADIAAAITACAAADSFNYKTFFAKCGLSAKSADEVKKAFFVIDQDKSGFIEEDELKLFLQTFKA
GARALTDAETKAFLAAGDADGDGMIGVDEFAALVKA

>Clupea_harengus_(Atlantic_herring)_pvalb2B
Sequence: GenBank XP_012694369
MAFAGILKDADIAAAIKDCAAADSFNYKTFFVKCGLSAKSSDEVKKAFFVIDQDKSGFIEEDELKFFLQTFKA
GARALTDAETKAFLAAGDEDGDGMIGVDEFAVLVKA

>Clupea_harengus_(Atlantic_herring)_pvalb3
Sequence: GenBank XP_012694365
MALASLLKGADIDAALKACEAKDSFKHKDFFAKIGLATKSAADLKKAFEIIDQDKSGFIEEEELKLFLQNFKA
GARALTDAETKAFLKAGDADGDGMIGVDEFSVMIKP

>Clupea_harengus_(Atlantic_herring)_pvalb4
Sequence: GenBank XP_031435403
MAFAAFLKEADITAALGACKGADSFDHKAFFAKVGLKGKSGDELKKAFEIIDQDKSGFIEEEELKLFLQNFCK
GARALTDGETKKFLKAGDSDNDGKIGIDEFTALIKH

>Clupea_harengus_(Atlantic_herring)_pvalb5
Sequence: GenBank XP_031426141
MAITGMLKEDDIRNAIEACQGPDTFDFKSFFKLAGLCSRPQADREKVFGVVDQDQSGFIEEAELKFFLQNFLL
GARELTEAETKSFLLAADCDGDGKIGMEEFCSLMN

Clupea_harengus_(Atlantic_herring)_pvalb6
Sequence: GenBank XP_031416779
MTMDSILSADDIKKALDSFKAAESFDHNKFFEMVGLKAKSDEEIKKAFLVLDADNSGFIEEEELKFVLKCFAP
DGRDLTDKETKAFLNAADKDGDGKIGVDEFTALVHE

>Clupea_harengus_(Atlantic_herring)_pvalb7
Sequence: GenBank XP_031419732
MAMKDLLTEENIKKAMDAFKAADSFDYKKFFDMVGLKAMSADSVKQVFKLLDVDESGYIEEEELKFVLKGFSS
EGRDLTDKETQTFLSAADKDGDGKIGIDEFEALVHE

>Clupea_harengus_(Atlantic_herring)_pvalb8
Sequence: GenBank XP_012681239
MSISSILSADAIDSAVKDCQAPDSFNCKKFFQLCGLTKKSAQDVKNVFSIIDNDGDGFVEEKELKCFLQKFSP
GARVLTEKEAKAFLSAADGDGDGKIGADEFQAMVMS

Clupea_harengus_(Atlantic_herring)_pvalb9
Sequence: GenBank XP_012694346
MSLTSILSAEAIDNAIKDCQDPDTFCYKKFFQLCGLSQKTPQEVKDVFRILDDDDSGFIEESELKFFLQQFVP
TARVLTEKEAKSFMAAADKDSDGKIGVDEFQSMILS

>Clupea_harengus_(Atlantic_herring)_pvalb10
Sequence: Modification of GenBank XP_012694332
MAVKDLFAASDITSAISSCQANDSFSPKAFFATVGLSKKSPPEIEKVFRILDQDKSGFIEQEELQLFLQNFSK
GARTLTAAETRAFMLDGDMDGDGKIGWEEFSALVKSS

Ictalurus punctatus (channel catfish)

>Ictalurus_punctatus_(channel_catfish)_pvalb1
Sequence: GenBank XP_017348365
MAFAGVLADADVTAAIAACSAPDSFDYKAFFAKVGLSAKSADDVKKAFSIIDQDNSGFIEEDELKLFLQNFKS
GARALTDKETKAFLAAGDSDGDGKIGAEEFAALVKA

>Ictalurus_punctatus_(channel_catfish)_pvalb2
Sequence: GenBank NP_001187965
MAFAGILKDEDVAAAISACSAADSFNYKTFFAKVGLSAKSADEIKKAFFVIDQDKSGFIEEDELKLFLQNFSA
GARALTDAETKAFLAAGDSDGDGKIGVDEFAALVKA

>Ictalurus_punctatus_(channel_catfish)_pvalb3
Sequence: GenBank XP_017338080
MAFAGLLSDADITAALQACQAADSFNYKTFFSKVGLSGKSSDDVKKAFAVIDQDKSGFIEEDELKLFLQNFSA
GARALTDAETKAFLAAGDSDGDGKIGVDEFAALVKA

>Ictalurus_punctatus_(channel_catfish)_pvalb4
Sequence: GenBank AAO25757
MAFAGVLNDADITAALDACKADGSFNHKSFFTKVGLTGKSADDVKKAFAIIDQDKSGFIEEDELKLFLQNFKS
SARALTDAETKTFLKAGDTDGDGKIGVDEFASLVKA

>Ictalurus_punctatus_(channel_catfish)_pvalb5

Sequence: GenBank XP_017348340
MAFAGILKEEDIAAAVQACLDPGTFCHNSFFEQVGLFSNSNVDGEKVFKVLDQDKSGFIEEEELKLFLQNFVA
GARELTDAETKAFLAAGDSDGDGKIGMQEFCALLN

>Ictalurus_punctatus_(channel_catfish)_pvalb6
Sequence: GenBank XP_017312511
MAMSSILNADDIKKALDTFKAADSFDHKKFFEMVGLKAKSADDVKKAFHVLDADNSGFIEEEELKFVLKGFAT
NGRDLTDKETKAFLKAADKDGDGKIGVEEFAALVHE

>Ictalurus_punctatus_(channel_catfish)_pvalb7
Sequence: GenBank NP_001187453
MAMQDLLKADNIKKALDTFKAADSFDHKKFFELVGLTAMSAEDVKKVFRVLDVDASGFIEEDELKFVLKGFSK
DGRDLTDKETKAFLTAADKDGDGKIGIDEFQAIVHQ

>Ictalurus_punctatus_(channel_catfish)_pvalb8
Sequence: GenBank NP_001188142
MSLTSILSADAIDSAIKDCQAPESFNHKKFFQLCGLSKKTPQEIRTVFGILDNDGSGFIEEEELKFFLQRFSA
GARVLTERETKTLLSAADDDGDGMIGAEEFQSMVVS

>Ictalurus_punctatus_(channel_catfish)_pvalb9
Sequence: GenBank XP_017338324
MSLTDILSAEAIENAIKDCEAPDSFSYKKFFQVIGLTKKSPEEVREVFRLLDDNNSGFIEESELKFFLRRFVP
GARTLTENETKNFMSGSDGNSDGKIGVDEFQSMVLS

>Ictalurus_punctatus_(Channel_catfish)_pvalb10
Sequence: GenBank XP_017338318
MAFTGFLAASDISSAINACKAKDSFSPKTFFATLGLSKKTPSEIEKVFKMLDQDQSGFIEQDELQLFLQNFSK
GARTLTAAEVKAFLVAGDMDGDGKIGWDEFSALVNDS

Esox lucius **(Northern pike)**

>Esox_lucius_(Northern_pike)_pvalb2A
Sequence: Encoded by Ensembl dataset Eluc_v4 transcript pvalb2-204
MSFAGLKDADVAAALAACSAADSFKHKEFFAKVGLASKSLDDVKKAFYVIDQDKSGFIEEDELKLFLQNFSPS
ARALTDAETKAFLADGDKDGDGMIGVDEFAAMIKA

>Esox_lucius_(Northern_pike)_pvalb2B
Sequence: Encoded by Ensembl dataset Eluc_v4 transcript pvalb2-203
MAFKGMLKDEDVAAALQAFAAADSFNHKQFFAKVGLAGKSDEDLKKAFYLVDQDKSGFIEEEELKLFLQTFSA
GARALTDTETKAFLAAGDVDGDGMIGVDEFVTLVKA

>Esox_lucius_(Northern_pike)_pvalb4
Sequence: Encoded by Ensembl dataset Eluc_v4 transcript pvalb4-201
MACAHLCKEADIKTALEACKAPESFNFTAFFKAVGFASKTADEVKKAFQVIDQDASGFIEEEELKLFLQNFCP
NARTLTDAETKAFLKAGDVDGDGMIGIDEFAELVKQ

>Esox_lucius_(Northern_pike)_pvalb6
Sequence: Encoded by Ensembl dataset Eluc_v4 transcript pvalb6-201
MAMSSILNAADIKKALDAFAAEGSFDHKKFFEMVGLKAKSAEDVKKAFLVLDADASGFIEEEELKFVLKGFAS
DGRDLTDKETKAFLNEADKDGDGMIGIDEFVALVHE

>Esox_lucius_(Northern_pike)_pvalb7
Sequence: Encoded by Ensembl dataset Eluc_v4 transcript pvalb7-202
MAAKDLLKADDIKKALDAVKAEGSFDHKKFFALVGLKAMSADNVKKVFKAIDADASGFIEEEELKFVLKSFAA
DGRDLTDAETKAFLKAADKDGDGKIGIDEFETLVHEA

>Esox_lucius_(Northern_pike)_pvalb8
Sequence: Encoded by Ensembl dataset Eluc_v4 transcript pvalb8-201
MSLSSILCTDAIETALKECLAPDSFNCRKFFQQCGLSKKSPAEVKTVFKILDNDNSGYIEEEELKYILQRFSL
GARVLTDKETKAFMSACDDDSDGMIGADEFQAMVLS

>Esox_lucius_(Northern_pike)_pvalb9
Sequence: Encoded by Ensembl dataset Eluc_v4 transcript pvalb9-201
MSLTSILSAEAIENAVKEFQAPESFSFKKFFQLCGLSSKSPKEVKDVFQILDSDNSGYIEEAELKFFLQRFVP
GARTLTDTECKGFLSAADDDNDGKIGVEEFLTMVQS

Gadus morhua **(Atlantic cod)**

>Gadus_morhua_(Atlantic_cod)_pvalb1
Sequence: GenBank XP_030204978
MSFAGVLADADVKAALAGCAAADSFSYKTFFKACGLAAKSHEEVKKAFFVIDQDQSGFIEEDELKLFLQTFGA
GARELTAAETKAFLAAGDVDGDGMIGVDEFVTLVKA

>Gadus_morhua_(Atlantic_cod)_pvalb2
Sequence: GenBank XP_030196388
MAFAGILADADCAAAVKACEAAESFSYKAFFAKCGLSGKSADDIKKAFFVIDQDKSGFIEEDELKLFLQVFKA
GARALTDAETKAFLKAGDSDGDGAIGVDEWAVLVKA

>Gadus_morhua_(Atlantic_cod)_pvalb3
Sequence: GenBank XP_030196389
MAFAGILNDADITAALAACKAEGSFDHKAFFTKVGLAAKSPADIKKVFEIIDQDKSDFVEEDELKLFLQNFSA
GARALSDAETKVFLKAGDSDGDGKIGVDEFGAMIKA

>Gadus_morhua_(Atlantic_cod)_pvalb4
Sequence: GenBank XP_030205636
MAFAGILKDAEVAAALEACKSAGSFDHTKFFKSCGLAGKSSDDVKKAFGIIDQDQSDFIEEEELKLFLQNFSA
SARALSDAETKAFLKAGDSDGDGKIGVDEFAAMVKA

>Gadus_morhua_(Atlantic_cod)_pvalb6
Sequence: GenBank XP_030198131
MAMTSILSADDIKKALNAFAAADSFDHKKFFEVVGLKALSAEEVKKAFLVLDADNSGFIEEEELKFMLKGFSS
SGRDLTDQETKAFLNAADKDGDGKIGVEEFTALVKE

>Gadus_morhua_(Atlantic_cod)_pvalb7
Sequence: GenBank XP_030208758
MAMTDLLKAEDIKKALDAFAVADSFDHRKFFELVGMRAMEADSVNKVFQALDVDASGYIEEEELKFVLKGFSE
EGRDLTDAETKKFLDAADKDGDGKIGMDEFQALVHE

>Gadus_morhua_(Atlantic_cod)_pvalb8

Sequence: GenBank XP_030203868
MSLSSILSADAIDNAIKDCQAPDSFCPKKFFQICGLSKKSPAEVKKAFAIMDNDASGFIEEEELKFFLQRFSP
GARVLTAAETKNFLSAADDDSDGKIGADEFQSLVLA

>Gadus_morhua_(Atlantic_cod)_pvalb9
Sequence: GenBank XP_030196391
MSLTSILSAEAIESAVKDCQEPNSFCYKKFFKLCGLTSKSPQEVKDVFSILDEDNSGYIEESELKFFLQRFVP
GARTLTEAEAKSFVSAADDDNDGKIGVEEFQTMVQS

>Gadus_morhua_(Atlantic_cod)_pvalb10
Sequence: GenBank XP_030196982
MAITDFLAASDITSAISACKAKDSFSAKMFFKTVGLSKKTAAEVEKVFKILDQDKSGFIEQDELQLFLQNFSK
GARNLTAAETRAFLLAGDSDGDGKIGWEEFSALVKSS

***Takifugu rubripes* (Fugu)**

>Takifugu_rubripes_(Fugu)_pvalb1
Sequence: GenBank XP_011602188
MAFAGILNDADVKAALDSCSAADSFNYKSFFQKCGLAAKSPDDLKKAFAIIDQDNSGFIEEEELKLFLQNFSA
SARALSDKETKAFLAAGDSDGDGKIGVEEFAALMKA

>Takifugu_rubripes_(Fugu)_pvalb3
Sequence: GenBank XP_011602756
MAFSGILSDADISAALAACQAADSFNHKSFFAKVGLAGKSKEDIAKAFGIIDQDKSGFIEEDELKLFLQNFSA
SARALTDTETKAFLKAGDTDGDGKIGVDEFAALVK

>Takifugu_rubripes_(Fugu)_pvalb4
Sequence: Modification of GenBank XP_011602190
MAFSGTLKDADVTAALEACKADGTFEYKKFFATCGLASKSGEELKKAFEIIDQDKSGFIEEEELKLFLQNFKA
GARALTDAETSAFLKAGDTDGDGKIGAQEFSDMVKA

>Takifugu_rubripes_(Fugu)_pvalb5
Sequence: GenBank XP_003964605
MAFAEMLSDENINAAVQACHAPGTFDLDSFFTQVGLSGSSQDVGEKVFTVLDQDKSGYIEEEELKRFLQNFSP
GARELTVAETKAVMAVLDKDGDGKIGMEEFCDILK

>Takifugu_rubripes_(Fugu)_pvalb6
Sequence: GenBank XP_029692838
MAMSSILNNDDIKKALDAFAVADSFDHKKFFEMVGLKSKSPDEVKKVFTVLDADNSGFIEEEELKFVLKGFAK
EGRDLTDKETKAFLQAADKDGDGKIGVDEFTSLVKE

>Takifugu_rubripes_(Fugu)_pvalb7
Sequence: Encoded by Ensembl dataset fTakRub1.2 transcript ENSTRUT00000061856
MLMTDLLKAEEIKTALEAFAGETFDPKKFFELVGLTAMSPESVKDVFRVLDVDGSGFIEEDELKYILKGFSKE
GRDLTDDETKAFLKAADKDGDGKIGIDEFEVMVHE

>Takifugu_rubripes_(Fugu)_pvalb8
Sequence: GenBank XP_003964606

MSLSSILPGDAIESAIKACQAPDSFCPKKFFQMCGLSKKSPQDIKKVFAILDNDGSGYIEEEELKFFLQRFCT
GARVLTDKETKGFMTLADDDSDGRIGADEFQAMVSS

>Takifugu_rubripes_(Fugu)_pvalb9
Sequence: GenBank XP_003961387
MSLTSILSAEAIENAVKDCQAPESFCYKRFFQLCGLSSKTPKEIQDVFHILDDDDSGYFEESELKYFLQRFQP
GARTLTEAETKSFISAADDDCDGRIGVEEFQSMVLS

>Takifugu_rubripes_(Fugu)_pvalb10
Sequence: GenBank XP_003961539
MAIANFLTASDITSAINACKAKDSFNPSVFFKTIGLSKKTPAEIETVFKILDQDKSGYIEQDELQLFLQNFSK
GARPLTAAETRAFLMEGDSDGDGKIGWDEFSALVKSS

Seriola lalandi (Yellowtail amberjack)

>Seriola_lalandi_(Yellowtail_amberjack)_pvalb1
Sequence: GenBank XP_023262140
MAFASVLKEAEVKAALDGCSAADSFNYKTFFKACGLSGKTAEDVKKAFAIIDQDNSGFIEEEELKLFLQNFSA
GARALTDKETKAFLAAGDSDGDGKIGVDEFAALVKA

>Seriola_lalandi_(Yellowtail_amberjack)_pvalb3
Sequence: GenBank XP_023285078
MAFAGVLSDADITAALAACQAADSFKHKDFFAKVGLAAKSADDIKKAFAIIDQDKSGFIEEEELKLFLQNFSA
GARALTDAETKAFLKAGDSDGDGKIGVDEFAAMVKH

>Seriola_lalandi_(Yellowtail_amberjack)_pvalb4
Sequence: GenBank XP_023250942
MAFKGVLNDADITAALDGCKNADSFDHKKFFKACGLAGKSADEVKKAFAIIDQDKSGFIEEEELKLFLQTFKA
GARALTDAETKTFLKAGDTDGDGKIGVDEFAAMVKH

>Seriola_lalandi_(Yellowtail_amberjack)_ pvalb5
Sequence: GenBank XP_023250944
MAFAGMLSDEDIKAAVKACQAPGTFNFKSFFTQVGLTGSSEADGKKVFTVLDQDKSGYIEEEELKLFLQNFSP
GARELTVDETKSLMAVGDRDGDGKIGMEEFCDLLK

>Seriola_lalandi_(Yellowtail_amberjack)_ pvalb6
Sequence: GenBank XP_023251550
MAMSSILNADDIKKALDAFAVADSFDHKKFFEMVGLKAKSCDDVKKVFTVLDADNSGFIEEEELKFVLKGFAK
DGRDLTDKETKAFLRAADKDGDGKIGVDEFAALVKE

Seriola_lalandi_(Yellowtail_amberjack)_pvalb7
Sequence: GenBank XP_023286972
MSMTDLLKAEEIKKALDAFAAETFDPKKFFEMVGMKAMTAENVKKVFQVLDVDGSGFIEEEELKFVLKGFSKE
GRDLTDAETKAFLQAADKDGDGKIGIDEFETMVHE

>Seriola_lalandi_(Yellowtail_amberjack)_pvalb8

Sequence: GenBank XP_023250929
MSLSSILSADAIDSAIKDCQAPDSFCPKKFFQLCGLTKKSPQDVKKVFAILDNDASGFVEEEELKFFLQRFSP
GARVLTDTETKAFLCAADDDSDGRIGADEFQAMVLS

>Seriola_lalandi_(Yellowtail_amberjack)_ pvalb9
Sequence: GenBank XP_023285083
MSLTSILSAEAIENAVKDCQAPDSFCYKKFFQLCGLSSKTPKEVKDVFQILDEDNSGFIEESELKYFLQRFIP
GARTLTEAETKSFISAADDDSDGRIGAEEFQTMVLS

>Seriola_lalandi_(Yellowtail_amberjack)_pvalb10
Sequence: GenBank XP_023285072
MAITDFLAASDITSAINACKAKDSFSPKMFFKTVGLSKKSPTEIERVFKILDQDKSGYIEQDELQLFLQNFAK
GARPLTAAETRAFLLEGDSDGDGKIGWEEFSALVKSS

Seriola dumerili (greater amberjack)

>Seriola_dumerili_(greater amberjack)_pvalb1
Sequence: GenBank XP_022608146
MAFASVLKEAEVKAALDGCSAADSFDYKTFFKACGLAGKTAEDVKKAFAIIDQDNSGFIEEEELKLFLQNFSA
GARALTDKETKAFLAAGDSDGDGKIGVDEFAALVKA

>Seriola_dumerili_(greater amberjack)_pvalb3
Sequence: GenBank XP_022598222
MAFAGVLSDADITAALAACQAADSFKHKDFFAKVGLAAKSADDIKKAFAIIDQDKSGFIEEEELKLFLQNFSA
GARALTDAETKTFLKAGDSDGDGKIGVDEFAAMVKH

>Seriola_dumerili_(greater amberjack)_pvalb4
Sequence: GenBank XP_022608144
MAFKGVLNDAEITAALDACKNADSFDHKKFFKACGLAAKSADEVKKAFAIIDQDKSGFIEEEELKLFLQTFKA
GARALTDAETKTFLKAGDTDGDGKIGVDEFAAMVKH

>Seriola_dumerili_(greater amberjack)_pvalb5
Sequence: GenBank XP_022608145
MAFAGMLSDEDIKAAVKACQAPGTFNFKSFFTQVGLTGSSEADGKKVFTVLDQDKSGYIEEEELKLFLQNFSP
GARELTVAETKSLMAAGDRDGDGKIGMEEFCDLLK

>Seriola_dumerili_(greater amberjack)_pvalb6
Sequence: GenBank XP_022612726
MAMSSILNADDIKKALDAFAVADSFDHKKFFEMVGLKAKSCDDVKKVFTVLDADNSGFIEEEELKFVLKGFAK
DGRDLTDKETKAFLRAADKDGDGKIGVDEFAALVKE

>Seriola_dumerili_(greater amberjack)_pvalb7
Sequence: GenBank XP_022593842
MSMTDLLKAEEIKKALDAFAAETFDPKKFFEMVGMKAMTAENVKKVFQVLDVDGSGFIEEEELKFVLKGFSKE
GRDLTDAETKAFLQAADKDGDGKIGIDEFEAMVHE

>Seriola_dumerili_(greater amberjack)_pvalb8
Sequence: GenBank XP_022608123

MSLSSILSADAIDSAIKDCQAPDSFCPKKFFQLCGLTKKSPQDVKKVFAILDNDASGFVEEEELKFFLQRFSP
GARVLTDTETKAFLCAADDDSDGRIGADEFQAMVLS

>Seriola_dumerili_(greater amberjack)_pvalb9
Sequence: GenBank XP_022598299
MSLTSILSAEAIENAVKDCQAPDSFCYKKFFQLCGLSSKTPKEVKDVFQILDEDNSGFIEESELKYFLQRFIP
GARTLTEAETKSFISAADDDSDGRIGAEEFQTMVLS

>Seriola_dumerili_(greater amberjack)_pvalb10
Sequence: GenBank XP_022598278
MAITDFLAASDINSAINACKAKDSFSPKMFFKTVGLSKKSPTEIERVFKILDQDKSGYIEQDELQLFLQNFAK
GARPLTAAETRAFLLEGDSDGDGKIGWEEFSALVKSS

Scophthalmus maximus (turbot)

>Scophthalmus_maximus_(turbot)_pvalb1
Sequence: GenBank
XP_035472137MSFAGLNAAEIKTALDGCAAADTFNYKKFFGACGLAKKSADDVKAAFQIIDQDNSGFIEEE
ELKLFLQNFSAGARALTDKETAEFLKAGDADGDGKIGIEEFANLVKE

>Scophthalmus_maximus_(turbot)_ pvalb3
Sequence: GenBank XP_035469716
MAFGKILSEAEITAALAACQAADSFKHKEFFAKVGLSAKSAEQIKEAFYVIDQDKSGFIEEEELKLFLQAFSA
SARALTDTETKEFLKAGDVDGDGKIGIDEFAEMVKQ

>Scophthalmus_maximus_(turbot)_pvalb4
Sequence: GenBank XP_035472139
MAFKGVLDDAKITAALEECKKPESFCHKKFFGTCGLSAKSPADVKAAFDIIDQDKSGFIEEDELKLFLQTFKA
GARSLTDTETKNLLKAGDTDNDGKIGADEFASMVKV

>Scophthalmus_maximus_(turbot)_pvalb5
Sequence: GenBank XP_035472138
MAFAGMLNEEDIKAAVQACQAPGTFNFKSFFAQVGLTGSTVADGEKVFTVLDQDRSGYIEEEELKLFLQNFSP
GARELTVAETKTLMASGDKDSDGKIGMEEFCDLLK

>Scophthalmus_maximus_(turbot)_pvalb6
Sequence: GenBank XP_035470782
MAMSSILNTDDIKKALHAFAAADSFDHKKFFEMVGLKAKPFDDVRKVFTVLDADNSGFIEEEELKYVLKGFAK
DGRDLTDKETQKFLKAADKDGDGKIGVDEFAALVKE

>Scophthalmus_maximus_(turbot)_pvalb7
Sequence: GenBank XP_035492367
MTMTDLLKAEEIKNALEAFAAETFDHRKFFEMVGMRAMSAENVKKVFQVLDVDGSGFIEEEELKFVLKGFSQE
GRDLTEAETKEFLKAADKDGDGKIGIDEFEAMVHE

>Scophthalmus_maximus_(turbot)_pvalb8
Sequence: GenBank XP_035472136
MSLSSILSADAIASAIKDCQAPDSFSPKKFFELSGLSKKSAQDVKKVFGILDNDASGFIEEDELKFFLQRFSP
GARVLTDKETKAFLSAADDDSDGRIGAEEFQAMILS

>Scophthalmus_maximus_(turbot)_pvalb9

Sequence: GenBank XP_035469767
MSLTSILSAEAIEKAVMDCQVPDSFCHKKFFSLCGLSSKTPKEVQDVFQILDDDKSGFIEESELKYFLQRFVP
GARTLTEAETKSFISAADGDSDGKVGVEEFRSLVQS

>Scophthalmus_maximus_(turbot)_pvalb10
Sequence: GenBank XP_035469765
MAVTDFLAASDVTLAINACKAKDSFSPKTFFKTVGLSKKSPKEIERVFKILDQDKSGFIEQDELQLFLQNFAK
GARPLTAAETRAFLMEGDSDGDGKIGWEEFSALVKST

Sparus aurata (gilthead seabream)

>Sparus_aurata_(gilthead_seabream)_pvalb1
Sequence: GenBank XP_030263619
MAFAGVLKDAEVKAALDGCSAADSFNYKSFFKACGLSGKSSDEVKKAFAIIDQDNSGFIEEDELKLFLQNFVA
SARALTDKETKAFLAAGDSDGDGKIGVDEFAALVKA

>Sparus_aurata_(gilthead_seabream)_pvalb3
Sequence: GenBank XP_030255186
MAFAGLLKEADITAALAACQAADSFKHKDFFAKVGLAGKSADDIKKAFAVIDQDKSGFIEEDELKLFLQNFSA
SARALTDGETKTFLKAGDSDGDGKIGVDEFAAMMKA

>Sparus_aurata_(gilthead_seabream)_pvalb4
Sequence: GenBank XP_030262240
MPFAGLTDADVAAALDGCKDAGSFDHKKFFAACGLSKKSGDDVKKAFAVIDQDKSGFIEEEELKLFLQNFKAG
ARALTDDETKKFLKAGDTDGDGKIGVDEFAAMVKV

>Sparus_aurata_(gilthead_seabream)_pvalb5
Sequence: GenBank XP_030263620
MAFAGMLSDEDVKAAVQACQTPGTFDFKTFFARVGLTGSSEADGKRVFTILDQDRSGYIEEEELKLFLQNFSP
EARELTVAETKALMAAGDKDGDGKIGLEEFCDILK

>Sparus_aurata_(gilthead_seabream)_pvalb6
Sequence: GenBank XP_030264822
MAMSSILNADDIKKALDAFAAADSFDYRKFFDMVGLKAKSSDDVKKVFKVLDADNSGFIEEEELKFVLKGFAK
DGRDLTDKETKAFLNAADKDGDGKIGVDEFVQLVTE

>Sparus_aurata_(gilthead_seabream)_pvalb7
Sequence: GenBank XP_030272489
MSMTDLLKAEEIKKALDAFAAETFDPKKFFEMVGLKAMTAENVKRVFKVLDVDGSGFIEEEELKYVLKGFSQE
GRDLTDDETRAFLKAADKDGDGKIGIDEFEVLVHE

>Sparus_aurata_(gilthead_seabream)_pvalb8
Sequence: GenBank XP_030262580
MSLSSILSADAIDSAIKDCQAPDTFCPKKFFQACGLTQKSPQDVKKAFAILDNDGSGFIEEEELKFFLQRFSP
GARVLTDKETKGFLAAADDDSDGRIGAEEFQAMVLS

>Sparus_aurata_(gilthead_seabream)_pvalb9
Sequence: GenBank XP_030255407
MSLTSILSAEAIEKAVKDCQAPDTFCYKKFFQLCGLSSKSPKEIKDVFQILDEDNSGFIEESELKYFLQRFVP
GARTLTEAETKRFISAADDDSDGRIGAEEFQTMVLS

>Sparus_aurata_(gilthead_seabream)_pvalb10

Sequence: GenBank XP_030255320
MAITDFLAASDITSAINACKGKDSFSPKMFFKTVGLSKKTPTEIEGVFKILDQDKSGFIEQDELQLFLQNFSK
GARTLTAAETRAFLLEGDSDGDGKIGWEEFSALVKSS

>Sparus_aurata_(gilthead_seabream)_pvalb-like
Sequence: GenBank XP_ XP_030256701
MEDDFRPQMKKMAVAMGASLTEQDIDHMPPGMTSQGIFHYSRFLEYMRQFKTSEQREEAIKKAFVMLDKDASG
YIEWNEIKYILSTVPTATPSAPLSDEEVEAMIQAVDTDGDGRISYREFSDMVKKEKELKK

Oreochromis niloticus (Nile tilapia)

>Oreochromis_niloticus_(Nile_tilapia)_pvalb1
Sequence: GenBank XP_003452874
MAFAGVLSDADVKAALDGCAAADSFDYKKFFKASGLAAKTADDVKKAFKIIDQDNSGFIEEEELKLFLQNFSA
GARALTDKETKAFLSAGDSDGDGKIGIDEFAALVKA

>Oreochromis_niloticus_(Nile_tilapia)_pvalb3
Sequence: GenBank XP_013129480
MAFAGILTDADITAALAACQAADSFKHKDFFAKVGLAGKSADDIKKAFAVIDQDKSGFIEEDELKLFLQNFSA
SARALTDAETKTFLKAGDSDGDGKIGVDEFAALVKS

>Oreochromis_niloticus_(Nile_tilapia)_pvalb4
Sequence: GenBank XP_003452873
MAFAGVLTDADIAAALDASKDAGTFDHKKFFKSCGLHGKSADDIKKAFAIIDQDKSGFIEEDELKLFLQNFKS
GARVLNDAETKAFLGAGDTDGDGKIGADEFVVLVKAT

>Oreochromis_niloticus_(Nile_tilapia)_pvalb5
Sequence: GenBank XP_005457817
MAFAGMLSDEDIQAAVQACQAPGTFNFKSFFEQVGLTGLSEADRRKVFTVLDQDKSGYIEEEELKLFLQNFSP
GARELTVAETKALMAAGDKDSDGKIGIEEFCDLLK

>Oreochromis_niloticus_(Nile_tilapia)_pvalb6
Sequence: GenBank XP_003443184
MAMSSILNTDDIKKALDAFAVADSFDHKKFFEMVGLKSKSADDVKKVFTVLDADNSGFIEEEELKFVLKGFAK
DGRDLTDKETKAFLKAADKDGDGKIGVDEFTALVKE

>Oreochromis_niloticus_(Nile_tilapia)_pvalb7
Sequence: GenBank XP_003459401
MVMTDLLKPEEIKKALDAFAAETFDPKKFFEMVGMKAMSAENVKKVFQVLDVDGSGFIEEEELKFVLKGFSKD
GRDLTDAETKAFLTAADKDGDGKIGIDEFEALVHE

>Oreochromis_niloticus_(Nile_tilapia)_pvalb8
Sequence: GenBank XP_003452872
MSLSSILSADAIDSAIKDCQAPDSFCAKKFFQMCGLTKKSPQDIKKVFGILDNDASGFIEEEELKFFLQRFCP
GARVLTDKETKAFLSAADDDSDGMIGADEFQAMVLS

>Oreochromis_niloticus_(Nile_tilapia)_pvalb9
Sequence: GenBank XP_005448188
MSLTSILSADDIQNAIKDCEAPDTFCYKKFFKLCGLSSKTPKEVKDVFQILDEDNSGYIEHSELKFFLQRFIP
SARTLTDAETKSFISAADDDSDGKIGVEEFQSMVLS

Oryzias latipes (Japanese medaka)

>Oryzias latipes _(Japanese_medaka_drRD)_pvalb1
Sequence: GenBank XP_004071520
MAFAGLNDADVKAALDGCAAADSFDYKKFFKACGLASKSADEVKKAFATIDQDNSGFIEEEELKLFLQNFSAG
ARALTDKETKTFLAAGDSDGDGKIGVDEFASLVKA

>Oryzias latipes _(Japanese_medaka_drRD)_pvalb3
Sequence: GenBank XP_011486186
MAFAGVLNDADITAALAACKAADSFKHKEFFAKVGLAAKSADEIKKAFGIIDQDQSGFIEEEELKLFLQNFAA
GARVLTDAETKTFLKAGDSDGDGKIGVDEFAAMVKG

>Oryzias latipes _(Japanese_medaka_drRD)_pvalb4
Sequence: GenBank XP_004071524
MAFAGVLNDADITKALDECKGADTFDYKKFFKTCGLAGKSADEVKKAFYIIDQDKSGFIEEDELKLFLQNFSA
GARVLSDGETKTFLKAGDVDGDGKIGADEFSALVKA

>Oryzias latipes _(Japanese_medaka_drRD)_pvalb5
Sequence: GenBank XP_004071522
MAFAGMLSEENIKAAVQACQAPGTFDFRSFFAQVGLIDSSEADRRKVFTVLDRDESGYIEEEELKLFLQNFFP
GARELTGAETKALIAAGDKDCDGKIGMDEFCDLLE

>Oryzias latipes _(Japanese_medaka_drRD)_pvalb6
Sequence: GenBank XP_023813489
MAMSSILNADDIKKALDAFAVADSFDHKKFFEILGLRAKSADDVKKVFTVLDADNSGYIEEEELKFVLKGFAK
DGRDLTDKETKAFLKAADKDGDGKIGVDEFTALVKE

>Oryzias latipes _(Japanese_medaka_drRD)_pvalb7
Sequence: GenBank XP_023814662
MVMTDLLKPEEIKKALDAFAAETFDPKKFFEMIGMRAMTAENVKKVFQVLDVDASGYIEEDELKFVLKGFSKD
GRDLTDAETKAFLQAADKDGDGKIGIDEFEALVHE

>Oryzias latipes _(Japanese_medaka_drRD)_pvalb8
Sequence: GenBank XP_020561005
MSLSSILSADAIDKAIKDCQAPDSFCPKKFFQICGLTQKSPQDIKKVFGILDNDASGYIEDEELKFFLQRFTP
SARVLTDKETKAFLVAADGDSDGRIGAAEFQTLVLS

>Oryzias latipes _(Japanese_medaka_drRD)_pvalb9
Sequence: GenBank XP_004080202
MSLSSILSAEAIENAVKDCQAPDSFSFKKFSQLCGLSSKTPQEIRDVFQILDEDNSGYIEESELKFFLQRFLP
GARTLTEAETKSFISAADDNSDGRIGVDEFQAMVLS

Other parvalbumin sequences

(The below sequences are listed in alphabetic order based on the species names)

Amphiuma means (two-toed amphiuma)

>Amphiuma_means_(two-toed_amphiuma)_pvalb-beta
Sequence: GenBank P02616
AITDILSAKDIEAALSSVKAAESFNYKTFFTKCGLAGKPTDQVKKVFDILDQDKSGYIEEDELQLFLKNFCSS
ARSLSNAETKAFLFAGDSDGDGKIGVDEFQALVRS

>Amphiuma_means_(two-toed_amphiuma)_pvalb-alpha
Sequence: GenBank P02626
SMTDVIPEADINKAIHAFKAGEAFDFKKFVHLLGLNKRSPADVTKAFHILDKDRSGYIEEEELQLILKGF
SKEGRELTDKETKDLLIKGDKDGDGKIGVDEFTSLVAES

Coryphaenoides rupestris (roundnose grenadier)

**>Coryphaenoides_rupestris_(Roundnose_grenadier)_pvalb1_(partial,_mayb
e_pseudogene)**
Sequence: Encoded by GenBank genomic sequence PIJE02000705
MAFEGLAEADVKAALAGCTAADSFNYKTFFAACGLAKKSDADVKKAFFVIDQDQSGFIEEDEL

>Coryphaenoides_rupestris _(Roundnose_grenadier)_pvalb4
Sequence: Encoded by GenBank genomic sequence PIJE02000705
MALRGILKDADVTAALDACKVAGSFDHKKFFSSCGLSSKSSADVKKAFGIIDQDKSDFIEEDELKLFLQNFSA
GARALSDAETKVFLQAGDTDGDGKIGVDEFAAMVKA

>Coryphaenoides_rupestris_(Roundnose_grenadier)_pvalb5
Sequence: Encoded by GenBank genomic sequence PIJE02000705
MTFTEMLKDEDVKAAVQACQAPGTFDHKAFFEQVGLSGSAEDMAERVFTALDQDKSGYIEEEELKTSSPGARV
LTVAETKALLAAGDKDGDGKIGMAEFCALLK

**>Coryphaenoides_rupestris_(Roundnose_grenadier)_pvalb8_(maybe_pseudog
ene)**
Sequence: Encoded by GenBank genomic sequence PIJE02000705
MSLSSILSADAIDNAIKDCQAPRSSLKLCGLSKKSAADVKKAFSIMDNDASGFIEEEELKFFLQRFSPGARVL
TASETKAFLNAADDDSDGKIGADEFQSLVLS

Ctenopharyngodon Idella (grass carp)

>Ctenopharyngodon_idella_(grass_carp)_pvalb4
Sequence: GenBank QCY53440
MAFAGILNDADIAAALEACKAADSFNHKAFFAKVGLSAKSGDDVKKAFAIIDQDKSGFIEEDELKLFLQNFKA
DARALTDAETKIFLKAGDSDGDGKIGVDEFAALVKA

Cyprinus carpio (common carp)

Cyprinus_carpio_(common _carp)_cyp_c 1.01_pvalb3_(Chr.B12)
Sequence: GenBank XP_018969314
MAFAGILNDADITAALQGCQAADSFDYKSFFAKVGLSAKTPDDIKKAFAVIDQDKSGFIEEDELKLFLQNFSA
GARALTDAETKAFLKAGDSDGDGKIGVDEFAALVKA

>Cyprinus_carpio_(common carp)_pvalb4(Chr.A3)
Sequence: GenBank XP_018966256
MAFAGVLNDADIAAALEACKAADSFNHKAFFAKVGLTSKSADDVKKAFAIIDQDKSGFIEEDELKLFLQNFKA
DARALTDGETKTFLKAGDSDGDGKIGVDEFTALVKA

>Cyprinus_carpio_(common carp)_pvalb4(Chr.B3)
Sequence: GenBank XP_018966236
MAFAGVLNDADITAALEACKAADSFNHKTFFAKVGLTSKSADDVKKAFAIIDQDKSGFIEEDELKLFLQNFKA
GARALTDGETKTFLKAGDSDGDGKIGVDEFTALVKA

Lates calcarifer (barramundi perch)

>Lates_calcarifer_(barramundi perch)_pvalb3
Sequence: GenBank XP_018528002
MAFAGILNEADITAALAACQAADSFKHKDFFVKVGLAGKSDDDVKKAFAVIDQDKSGFIEEDELKLFLQNFSA
SARALTDAETKEFLKAGDSDGDGKIGVDEFAALVKV

>Lates_calcarifer_(barramundi perch)_pvalb4
Sequence: GenBank XP_018526999
MAFSNVLSDSDVAAALDGCKDAGTFDHKKFFSACGLSNKTSDDVKKAFAIIDQDKSGFIEEEELKLFLQNFKA
DARVLTDVETSTFLKAGDTDGDGKIGADEFTALVKP

Mustelus griseus (spotless smooth-hound shark)_

>Mustelus_griseus_(spotless smooth-hound shark)_SPV-II
Sequence: GenBank 5ZH6
GSITTVLNATDIAKALAQCAGSFNHKTFFVTSGLTNKSDANLAKVFDILDQDRSGFIEVDELKLFLQNFSATA
RELDETETNAFLAAGDSDHDGKIGVDEFKAMVKA

>Mustelus_griseus_(spotless smooth-hound shark)_SPV-I
Sequence: GebBank 5ZGM
MPMTKVLKADDINKAVSAFKDPGTFDYKRFFQLVGLKGKSEAQVKEVFEILDKDQSGFIEEEELKSVLKGFSA
HGRDLSDTETKALLAAGDSDHDGKIGADEFAKMVAQA

Pelophylax lessonae (pool frog) pvalb-beta

>Pelophylax lessonae (pool frog) pvalb-beta
Sequence: GenBank CAC95152
MSITDIVSEKDIDAALESVKAAGSFNYKIFFQKVGLAGKSAADAKKVFEILDRDKSGFIEQDELGLFLQN
FRASARVLSDAETSAFLKAGDSDGDGKIGVEEFQALVKA

>Pelophylax lessonae (pool frog) pvalb-alpha
Sequence: GenBank CAC83046
MPMTDLLAAGDISKAVSAFAAPESFNHKKFFELCGLKSKSKEIMQKVFHVLDQDQSGFIEKEELCLILKG
FTPEGRSLSDKETTALLAAGDKDGDGKIGVDEFVTLVSES

Plecoglossus altivelis (ayu)

>Plecoglossus_altivelis_(ayu)_pvalb2
Sequence: Encoded by GenBank genomic sequence BNHK01000005
MAFAGILKDDDVAAAIKACAAADSFKYKEFFAKVGLSAKSADDVKKAFYVIDQDKSGFIEEDELKLFLQNFAA
GARALTDAETKAFLKAGDADGDGMIGVDEFAALVKA

>Plecoglossus_altivelis_(ayu)_pvalb3
Sequence: Encoded by GenBank genomic sequence BNHK01000005
MAFAGVLKDDDITAALAACKAADSFKYKDFFAKVGLASKSADDVKKAFGIIDQDKSGFIEEEELKLFLQNFAA
GARALTDAETKAFLKAGDADGDGMIGMDEFAALVKS

Salmo salar (Atlantic salmon)

>Salmo_salar_(Atlantic_salmon)_pvalb4_(Chr.3)
Sequence: GenBank NP_001117190
MACAHLCKEADIKTALEACKAADTFSFKTFFHTIGFASKSADDVKKAFKVIDQDASGFIEVEELKLFLQNFCP
KARELTDAETKAFLKAGDADGDGMIGIDEFAVLVKQ

>Salmo_salar_(Atlantic_salmon)_pvalb5_(Chr.3)
Sequence: GenBank XP_014049628
MAFTGLLREEDIRAAVKACQAPSTFNFKLFFAQVGLTGKPEAEGQRVFRVLDQDQSGYIEEEELKLFLQNFSS
GARELTDAETRTLLAAGDRDGDGKIGMEEFCALLK

>Salmo_salar_(Atlantic_salmon)_pvalb10_(Chr.28)
Sequence: GenBank NP_001134150
MALTDFLAASDITSAINACRANDSFSPKKFFAMVGLSKKSPPEIEKIFMILDQDKSGYIEQDELQLFLQNFSK
GARPLTAAETRAFLLAGDKDGDGKIGWDEFSNLVMSS

Sardinops melanostictus (crimson seabream)

>Sardinops_melanostictus_(crimson_seabream)_pvalb4
Sequence: GenBank BAF98921
MALAGLVKEADITAALEACKAADSFDHKAFFHKVGMSGKSADELKKSFAIIDQDKSGFIEEEELKLFLQNFCK
KARALTDGETKNFLKAGDTDGDGKIGIDNFNHLVKH

Scomber japonicus (Pacific mackerel)

>Scomber_japonicus_(Pacific_mackerel)_pvalb4
Sequence: GenBank ABJ98932
MAFASVLKDAEVTAALDGCKAAGSFDHKKFFKACGLSGKSTDEVKKAFAIIDQDKSGFIEEEELKLFLQNFKA
GARALSDAETKAFLKAGDSDGDGKIGIDEFAAMIKG

Triakis semifasciata (leopard shark)

>Triakis_semifasciata_(leopard_shark)_pvalb-alpha
Sequence: GenBank P30563
PMTKVLKADDINKAISAFKDPGTFDYKRFFHLVGLKGKTDAQVKEVFEILDKDQSGFIEEEELKGV
LKGFSAHGRDLNDTETKALLAAGDSDHDGKIGADEFAKMVAQA
