# Supplementary material for: Comprehensive Sequence Analysis of Parvalbumins in Fish and Their Comparison with Parvalbumins in Tetrapod Species
Source: Biology (Basel). 2022 Nov 25;11(12):1713. doi: 10.3390/biology11121713 (PMC9774829; doi:10.3390/biology11121713)
Supplement: Supplementary file 1 [file biology-11-01713-s001.zip › Supplementary file S2.pdf]

Supplementary File S2

Transcriptome expression analysis

Page 2 Figure legend

Page 3 (A) *Lepisosteus oculatus* (spotted gar)

(B) *Anguilla anguilla* (European eel)

Page 4 (C) *Paramormyrops kingsleyae* (elephant fish)

(D) *Danio rerio* (zebrafish)

Page 5 (E) *Onychostoma macrolepis* (a cyprinid fish)

(F) *Clupea harengus* (Atlantic herring)

Page 6 (G) *Ictalurus punctatus* (Channel catfish)

(H) *Esox lucius* (Northern pike)

Page 7 (I) *Gadus morhua* (Atlantic cod)

(J) *Oryzias latipes* (Japanese medaka)

Figure legend

Expression per tissue in different fish species as estimated by database transcriptome analysis. Single read archive (SRA) datasets in the NCBI database (https://www.ncbi.nlm.nih.gov/sra) were investigated for matching reads by tblastn similarity searches. A minimum score, depending on the length of the reads, was set as the threshold value for accepting identity in which minimal deviations from a perfect match were accepted to account for possible allelic differences and sequencing errors. The data were then calibrated by calculating the number of matching reads per 100 million reads. The names of each SRA dataset are provided in the tables. This on-site method is relatively easy but has a disadvantage that only a maximum of 5000 matches per SRA dataset could be found; if the number of matches were 5000 or higher, this is indicated by the symbol “>.” Tissue nomenclature follows the respective database reports.

(A) *Lepisosteus oculatus* (spotted gar)

From bioproject PRJNA255881. Threshold: tblastn score >63 bits.

Tissue<br>Dataset	Brain<br>SRX661015	Muscle<br>SRX661018	Gills<br>SRX661016	Heart<br>SRX661017	Liver<br>SRX661019	Kidney<br>SRX661020	Bones<br>SRX661021	Intestine<br>SRX661022	Embryo<br>SRX661023	Ovary<br>SRX661024	Testis<br>SRX661025
8560	195.5	>8417.5	15.2	171.1	274.4	590.3	>8445.9	130.9	>9842.5	119.2	18.4
8573	33.8	>8417.5	11.4	114.4	193.1	422.1	>8445.9	87.3	>9842.5	90.1	11.7
8585	7.7	85.9	0.0	0.0	0.0	0.0	265.2	0.0	>9842.5	7.3	0.0
8594	469.3	>8417.5	22.7	3.3	18.1	37.4	2145.3	21.8	1635.8	2.9	0.0
pvalb-α	369.5	>8417.5	18.9	166.7	225.6	183.8	8300.7	63.6	25.6	0.0	13.4
8552	16.9	3.4	0.0	0.0	3.6	20.2	0.0	0.0	830.7	16.0	0.0
8606	97.7	0.0	98.5	20.0	10.8	3.1	32.1	27.3	39.4	5.8	431.4

(B) *Anguilla anguilla* (European eel)

From bioproject PRJNA256923. Threshold: tblastn score >63 bits.

Tissue<br>Dataset	Brain<br>SRX666381	Muscle<br>SRX666384	Gills<br>SRX666382	Heart<br>SRX666383	Liver<br>SRX666385	Kidney<br>SRX666386	Bones<br>SRX666387	Intestine<br>SRX666388	Larvae<br>SRX666390	Ovary<br>SRX666380	Testis<br>SRX666389
pvalb1	8.5	>11467.9	1761.8	7.3	11.1	1.7	>7374.6	14.0	>6024.1	25.4	62.1
pvalb3	31.3	>11467.9	10162.3	43.9	55.6	3.4	>7374.6	56.0	>6024.1	88.8	151.1
pvalb4	75.3	>11467.9	>13089.0	124.4	127.8	0.0	>7374.6	218.0	>6024.1	99.0	148.3
pvalb5	29.8	6.9	0.0	0.0	0.0	0.0	2.9	6.0	18.1	0.0	7.1
pvalb6	1009.9	0.0	0.0	0.0	0.0	0.0	0.0	0.0	0.0	2.5	1.4
pvalb7	1082.4	0.0	0.0	0.0	0.0	0.0	0.0	0.0	0.0	5.1	4.2
pvalb8	17.0	0.0	149.2	0.0	0.0	0.0	0.0	4.0	13.3	0.0	189.3
pvalb9	2.8	0.0	0.0	0.0	0.0	0.0	5.9	0.0	9.6	86.3	8.5
pvalb10	356.5	0.0	5.2	0.0	0.0	0.0	2.9	0.0	0.0	0.0	7.1

(C) *Paramormyrops kingsleyae* (elephant fish)

From bioproject PRJNA417677. Threshold: tblastn score >63 bits.

Tissue	Brain	Spinal cord	Skeletal muscle	Gill	Kidney	Spleen	Gonadal tissue	Fin	Electric organ	Swim bladder
Dataset	SRX3376033	SRX3376023	SRX3376022	SRX3376025	SRX3376021	SRX3376019	SRX3376024	SRX3376029	SRX3376032	SRX3376020
pvalb1	59.1	592.4	>11961.7	173.8	101.0	37.2	136.4	11.3	28.4	28.0
pvalb2	104.8	383.2	>11961.7	229.9	86.2	48.8	6.8	14.1	17.5	17.2
pvalb3	37.6	423.9	>11961.7	371.7	76.4	23.3	15.9	8.5	26.2	10.8
pvalb5ψ	0.0	0.0	57.4	0.0	0.0	0.0	0.0	0.0	0.0	0.0
pvalb6	2032.3	65.2	52.6	10.7	2.5	0.0	0.0	0.0	0.0	0.0
pvalb7	86.0	206.5	1413.9	246.0	0.0	0.0	0.0	0.0	17.5	21.6
pvalb8	2.7	0.0	0.0	109.6	0.0	0.0	0.0	166.7	0.0	88.4
pvalb9	0.0	5.4	7.2	0.0	4.9	0.0	143.2	8.5	2.2	0.0

(D) *Danio rerio* (zebrafish)

From bioproject PRJNA721381. Threshold: tblastn score >63 bits.

Tissue	Brain	Muscle	Heart	Liver	Spleen	Intestine	Testis	Skin
Dataset	SRX10579896	SRX10579908	SRX10579899	SRX10579905	SRX10579914	SRX10579902	SRX10579920	SRX10579911
pvalb1	0.0	>32051.3	0.0	0.0	0.0	6.4	15.1	>26455.0
pvalb2	0.0	>32051.3	0.0	0.0	4.4	38.2	65.3	>26455.0
pvalb3	0.0	>32051.3	25.3	0.0	4.4	76.4	75.4	>26455.0
pvalb4	32.3	>32051.3	0.0	0.0	8.8	95.5	25.1	>26455.0
pvalb5	0.0	0.0	0.0	0.0	0.0	0.0	5.0	0.0
pvalb6	3574.2	6.4	0.0	14.4	13.2	25.5	16075.4	42.3
pvalb7	1574.2	2000.0	0.0	0.0	0.0	0.0	45.2	15650.8
pvalb8	77.4	737.2	0.0	0.0	0.0	0.0	15.1	381.0
pvalb9	19.4	0.0	6.3	0.0	0.0	1133.8	15.1	21.2

(E) *Onychostoma macrolepis* (a cyprinid fish)

From bioproject PRJNA608076. Threshold: tblastn score >93 bits.

Tissue	Brain	Muscle	Gill	Heart	Liver	Kidney	Spleen	Ovary	Testis
Dataset	SRX7828808	SRX7828814	SRX7828813	SRX7828810	SRX7828807	SRX7828812	SRX7828811	SRX7828815	SRX7828816
pvalb1	1.4	>6812.0	0.0	0.0	0.0	0.0	0.0	0.0	0.0
pvalb2	1.4	>6812.0	0.0	0.9	0.0	1.0	0.0	3.3	24.6
pvalb3	4.2	>6812.0	1.2	0.9	0.0	1.0	0.0	5.4	52.2
pvalb4	0.0	>6812.0	52.9	3.7	0.0	7.2	1.4	1.1	43.5
pvalb5	0.0	9.5	0.0	0.0	0.0	11.4	0.0	0.0	15.9
pvalb6	2135.7	0.0	19.2	0.9	1.2	673.9	42.6	35.9	608.7
pvalb7	3288.1	272.5	88.9	0.0	0.0	17.6	1.4	1.1	14.5
pvalb8	9.7	0.0	19.2	0.0	0.0	0.0	0.0	1.1	4.3
pvalb9	0.0	0.0	0.0	0.0	0.0	5176.0	0.0	1.1	115.9
pvalb10	0.0	1.4	2.4	1.9	1.2	0.0	0.0	1.1	0.0

(F) *Clupea harengus* (Atlantic herring)

From bioproject PRJEB31270. Threshold: tblastn score >63 bits.

Tissue	Brain	Muscle	Heart	Liver	Kidney	Spleen	Intestine	Eye
Dataset	ERX3290222	ERX3289287	ERX3291635	ERX3289464	ERX3289465	ERX3289214	ERX3290873	ERX3290261
pvalb1	1.3	>4310.3	10.9	0.3	9.2	1.7	0.8	1.2
pvalb2A	4.2	1175.9	0.0	0.0	83.5	0.6	0.8	0.2
pvalb2B	1.6	458.6	0.3	0.0	241.0	1.7	1.6	0.2
pvalb3	0.3	>4310.3	12.7	0.0	20.1	0.0	1.1	0.7
pvalb4	0.0	>4310.3	0.0	0.0	0.2	0.6	0.0	0.0
pvalb5	4.7	0.0	0.3	0.0	5.8	0.6	2.1	3.2
pvalb6	110.1	0.0	0.3	1.3	0.2	0.0	1.9	10.7
pvalb7	1112.5	5.2	4.2	0.8	0.5	4.2	9.4	15.8
pvalb8	2.1	0.0	0.3	0.0	0.2	0.0	0.0	39.7
pvalb9	28.7	0.0	0.0	0.0	0.7	1.4	0.8	783.2
pvalb10	727.6	0.9	0.0	3.8	0.0	1.4	0.8	1.7

(G) Ictalurus punctatus (Channel catfish).

From bioproject PRJNA432820. Threshold: tblastn score >63 bits.

Tissue	Spinal cord	Spinal cord	Spinal cord	Muscle	Muscle	Muscle
Dataset	SRX3651833	SRX3651842	SRX3651843	SRX3651824	SRX3651825	SRX3651832
pvalb1	125.4	116.7	31.1	>8116.9	>8012.8	>8090.6
pvalb2	91.3	435.0	57.4	>8116.9	>8012.8	7134.3
pvalb3	188.9	520.0	77.0	>8116.9	>8012.8	>8090.6
pvalb4	780.2	486.7	360.7	>8116.9	>8012.8	>8090.6
pvalb5	0.0	0.0	14.8	3.2	0.0	0.0
pvalb6	442.7	601.7	247.5	1.6	0.0	3.2
pvalb7	61.9	83.3	16.4	2952.9	4024.0	2799.4
pvalb8	0.0	0.0	0.0	0.0	0.0	3.2
pvalb9	0.0	0.0	0.0	0.0	0.0	0.0
pvalb10	0.0	0.0	0.0	3.2	1.6	0.0

(H) *Esox lucius* (Northern pike)

From bioproject PRJNA256965. Threshold: tblastn score >63 bits.

Tissue	Brain	Muscle	Gills	Heart	Liver	Kidney	Bones	Intestine	Embryo	Ovary	Testis
Dataset	SRX667247	SRX667250	SRX667248	SRX667249	SRX667251	SRX667252	SRX667253	SRX667254	SRX667255	SRX667246	SRX667256
pvalb2A	4.4	2876.7	0.0	3.3	3.8	0.0	315.1	3.6	>5071.0	0.0	2.9
pvalb2B	26.5	>8333.3	5.9	7.5	10.4	112.4	>13020.8	76.5	>5071.0	7.5	28.9
pvalb4	0.0	6178.3	0.0	2.5	0.0	0.0	1367.2	7.1	>5071.0	0.0	0.0
pvalb6	3372.8	0.0	8.9	0.8	0.0	1.7	596.4	3.6	88.2	0.0	28.9
pvalb7	996.7	>8333.3	8.9	0.8	0.9	25.2	>13020.8	35.6	9.1	2.5	20.2
pvalb8	11.1	0.0	0.0	0.0	0.0	0.0	0.0	0.0	73.0	0.0	1.4
pvalb9	771.0	0.0	4.4	0.0	0.0	0.0	0.0	0.0	101.4	7.5	33.2

(I) *Gadus morhua* (Atlantic cod)

From bioproject PRJNA256972. Threshold: tblastn score >63 bits.

Tissue	Brain	Muscle	Gills	Heart	Liver	Kidney	Bones	Intestine	Embryo	Ovary	Testis
Dataset	SRX1044006	SRX1044009	SRX1044007	SRX1044008	SRX1044010	SRX1044011	SRX1044012	SRX1044013	SRX1044015	SRX1044005	SRX1044014
pvalb1	1.4	2003.7	0.0	21.1	2.6	0.0	4.6	0.0	>7082.2	4.5	27.9
pvalb2	76.7	>9191.2	19.7	74.7	134.9	61.5	769.2	3.9	>7082.2	4.5	16.3
pvalb3	202.7	>9191.2	1.4	17.6	97.9	14.3	>7692.3	0.0	92.1	0.0	0.0
pvalb4	42.5	>9191.2	5.6	13.2	15.9	8.2	3927.7	0.0	3426.3	75.9	4.7
pvalb6	2817.8	5.5	5.6	51.8	21.2	65.6	181.5	0.0	464.6	0.0	0.0
pvalb7	>6849.3	3.7	0.0	0.0	0.0	0.0	4.6	0.0	29.7	0.0	4.7
pvalb8	230.1	34.9	98.6	0.0	21.2	6108.6	193.8	339.8	1582.2	58.0	11.6
pvalb9	8.2	0.0	38.0	0.0	0.0	0.0	0.0	46.9	69.4	4.5	7.0

(J) *Oryzias latipes* (Japanese medaka strain drRD)

From bioproject PRJDB12004. Threshold: tblastn score >49 bits.

Tissue	Forebrain	Midbrain	Hindbrain	Spinal cord	Muscle	Gill	Heart	Liver	Kidney	Bone	Spleen	Midgut
Dataset	DRX298708	DRX298748	DRX298728	DRX298764	DRX298756	DRX298720	DRX298724	DRX298744	DRX298736	DRX298688	DRX298768	DRX298752
pvalb1	3.2	6.0	4.4	2254.8	>13736.3	0.0	3.8	0.0	2.2	>26881.7	9.3	0.0
pvalb3	12.7	12.0	223.7	7454.3	>13736.3	0.0	0.0	2.6	0.0	>26881.7	0.0	8.9
pvalb4	44.3	48.2	447.4	8829.3	>13736.3	121.8	26.9	0.0	6.6	>26881.7	0.0	0.0
pvalb5	28.5	6.0	43.9	9.6	22.0	0.0	0.0	71.1	46.1	0.0	0.0	0.0
pvalb6	310.1	373.5	3302.6	1182.7	1310.4	37.8	0.0	0.0	4.4	6946.2	4.6	0.0
pvalb7	1417.7	1343.4	315.8	300.5	3033.0	937.0	0.0	0.0	2.2	6591.4	0.0	0.0
pvalb8	224.7	192.8	982.5	0.0	8.2	0.0	0.0	0.0	17.5	134.4	0.0	0.0
pvalb9	31.6	30.1	184.2	0.0	0.0	25.2	0.0	0.0	6.6	32.3	0.0	3.0
