# Supplementary material for: Comprehensive Sequence Analysis of Parvalbumins in Fish and Their Comparison with Parvalbumins in Tetrapod Species
Source: Biology (Basel). 2022 Nov 25;11(12):1713. doi: 10.3390/biology11121713 (PMC9774829; doi:10.3390/biology11121713)
Supplement: Supplementary file 1 [file biology-11-01713-s001.zip › Supplementary file S4.pdf]

Supplementary File S4

Phylogenetic tree analysis

Page 1 Figure legends

Page 3 (A) Tree by Maximum Likelihood method

Page 4 (B) Tree by Neighbor-Joining method

Page 5 (C) Tree by Minimum Evolution method

Page 6 (D) Tree by UPGMA method

Figure legends

(A) Molecular Phylogenetic analysis by Maximum Likelihood method. The evolutionary history was inferred based on the JTT matrix-based model [1]. The tree with the highest log likelihood (-15686.03) is shown. The percentage of trees in which the associated taxa clustered together is shown next to the branches. Initial tree(s) for the heuristic search were obtained automatically by applying Neighbor-Join and BioNJ algorithms to a matrix of pairwise distances estimated using a JTT model, and then selecting the topology with superior log likelihood value. The tree is drawn to scale, with branch lengths measured in the number of substitutions per site. The analysis involved 209 amino acid sequences. All positions with less than 95% site coverage were eliminated. Evolutionary analyses were conducted in MEGA7 [2].

(B) The evolutionary history was inferred using the Neighbor-Joining method [3]. The optimal tree with the sum of branch length = 25.67950417 is shown. The percentage of replicate trees in which the associated taxa clustered together in the bootstrap test (500 replicates) are shown next to the branches [4]. The tree is drawn to scale, with branch lengths in the same units as those of the evolutionary distances used to infer the phylogenetic tree. The evolutionary distances were computed using the Poisson correction method [5] and are in the units of the number of amino acid substitutions per site. The analysis involved 209 amino acid sequences. All ambiguous positions were removed for each sequence pair. Evolutionary analyses were conducted in MEGA7 [2].

(C) The evolutionary history was inferred using the Minimum Evolution method [6]. The optimal tree with the sum of branch length = 25.15980363 is shown. The percentage of replicate trees in which the associated taxa clustered together in the bootstrap test (500 replicates) are shown next to the branches [4]. The tree is drawn to scale, with branch lengths in the same units as those of the evolutionary distances used to infer the phylogenetic tree. The evolutionary distances were computed using the Poisson correction method [5] and are in the units of the number of amino acid substitutions per site. The ME tree was searched using the Close-Neighbor-Interchange (CNI) algorithm [7] at a search level of 1. The Neighbor-joining algorithm [8] was used to generate the initial tree. The analysis involved 209 amino acid sequences. All ambiguous positions were removed for each sequence pair. Evolutionary analyses were conducted in MEGA7 [2].

(D) The evolutionary history was inferred using the UPGMA method [9]. The optimal tree with the sum of branch length = 24.98273800 is shown. The percentage of replicate trees in which the associated taxa clustered together in the bootstrap test (500 replicates) are shown next to the branches [4]. The tree is drawn to scale, with branch lengths in the same units as those of the evolutionary distances used to infer the phylogenetic tree. The evolutionary distances were computed using the Poisson correction method [5] and are in the units of the number of amino acid substitutions per site. The analysis involved 209 amino acid sequences. All ambiguous positions were removed for each sequence pair. There were a total of 109 positions in the final dataset. Evolutionary analyses were conducted in MEGA7 [2].

References

1. Jones D.T., Taylor W.R., and Thornton J.M. (1992). The rapid generation of mutation data matrices from protein sequences. Computer Applications in the Biosciences 8: 275-282.
2. Kumar S., Stecher G., and Tamura K. (2016). MEGA7: Molecular Evolutionary Genetics Analysis version 7.0 for bigger datasets.Molecular Biology and Evolution 33:1870-1874.
3. Saitou N. and Nei M. (1987). The neighbor-joining method: A new method for reconstructing phylogenetic trees. Molecular Biology and Evolution 4:406-425.
4. Felsenstein J. (1985). Confidence limits on phylogenies: An approach using the bootstrap. Evolution 39:783-791.
5. Zuckerkandl E. and Pauling L. (1965). Evolutionary divergence and convergence in proteins. Edited in Evolving Genes and Proteins by V. Bryson and H.J. Vogel, pp. 97-166. Academic Press, New York.
6. Rzhetsky A. and Nei M. (1992). A simple method for estimating and testing minimum evolution trees. Molecular Biology and Evolution 9:945-967.
7. Nei M. and Kumar S. (2000). Molecular Evolution and Phylogenetics. Oxford University Press, New York.
8. Saitou N. and Nei M. (1987). The neighbor-joining method: A new method for reconstructing phylogenetic trees. Molecular Biology and Evolution 4:406-425.
9. Sneath P.H.A. and Sokal R.R. (1973). Numerical Taxonomy. Freeman, San Francisco.

(A) ML tree

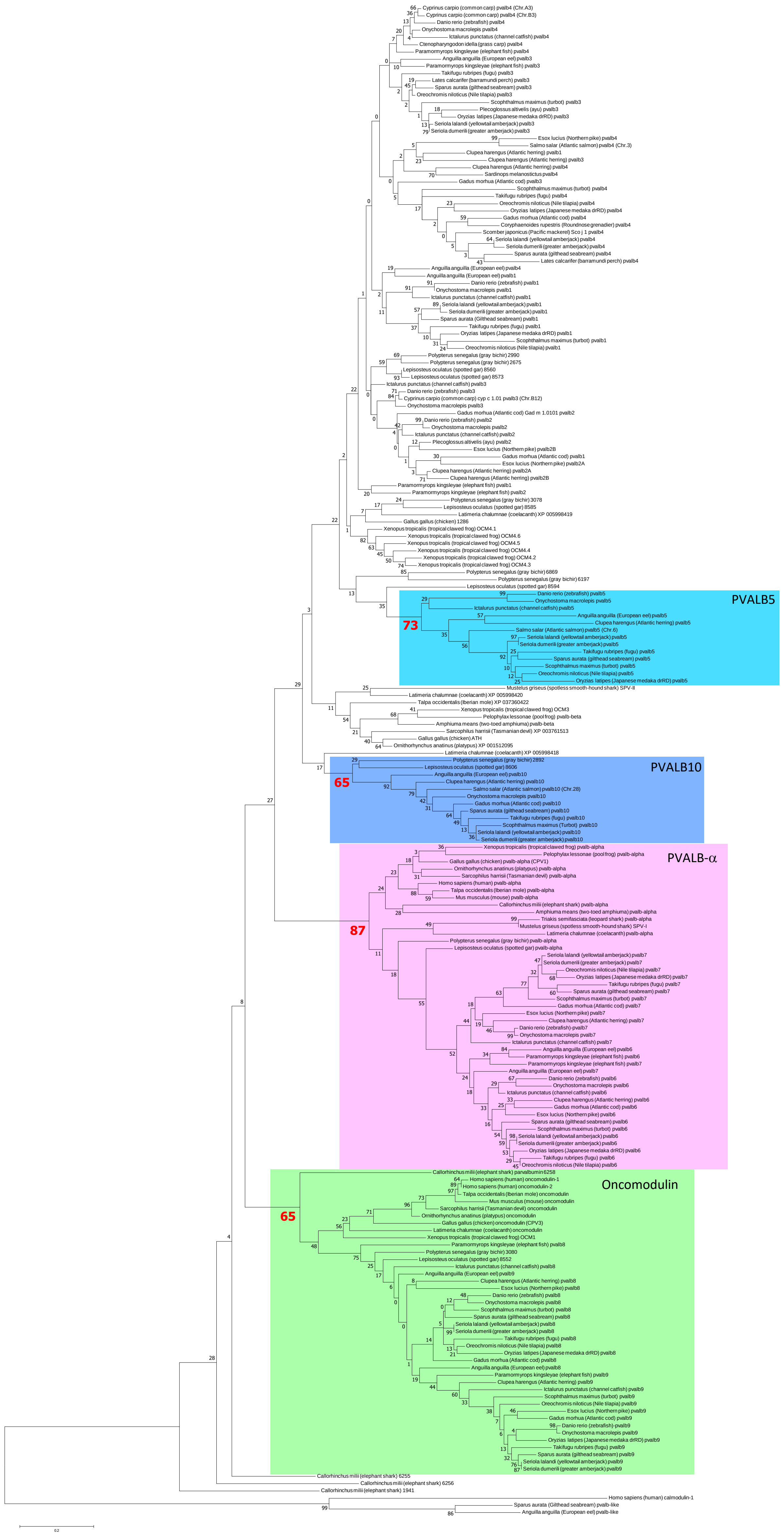

Cyprinus carpio (common carp) pvalb4 (Chr.A3)
Cyprinus carpio (common carp) pvalb4 (Chr.B3)
Danio rerio (zebrafish) pvalb4
Onychostoma macrolepis pvalb4
Ictalurus punctatus (channel catfish) pvalb4
Ctenopharyngodon idella (grass carp) pvalb4
Paramormyrops kingsleyae (elephant fish) pvalb4
Anguilla anguilla (European eel) pvalb3
Paramormyrops kingsleyae (elephant fish) pvalb3
Takifugu rubripes (fugu) pvalb3
Lates calcarifer (barramundi perch) pvalb3
Sparus aurata (gilthead seabream) pvalb3
Oreochromis niloticus (Nile tilapia) pvalb3
Scophthalmus maximus (turbot) pvalb3
Plecoglossus altivelis (ayu) pvalb3
Oryzias latipes (Japanese medaka drRD) pvalb3
Seriola lalandi (yellowtail amberjack) pvalb3
Seriola dumerili (greater amberjack) pvalb3
Esox lucius (Northern pike) pvalb4
Salmo salar (Atlantic salmon) pvalb4 (Chr.3)
Clupea harengus (Atlantic herring) pvalb1
Clupea harengus (Atlantic herring) pvalb3
Clupea harengus (Atlantic herring) pvalb4
Sardinops melanostictus pvalb4
Gadus morhua (Atlantic cod) pvalb3
Scophthalmus maximus (turbot) pvalb4
Takifugu rubripes (fugu) pvalb4
Oreochromis niloticus (Nile tilapia) pvalb4
Oryzias latipes (Japanese medaka drRD) pvalb4
Gadus morhua (Atlantic cod) pvalb4
Coryphaenoides rupestris (Roundnose grenadier) pvalb4
Scomber japonicus (Pacific mackerel) Sco j 1 pvalb4
Seriola lalandi (yellowtail amberjack) pvalb4
Seriola dumerili (greater amberjack) pvalb4
Sparus aurata (gilthead seabream) pvalb4
Lates calcarifer (barramundi perch) pvalb4
Anguilla anguilla (European eel) pvalb4
Anguilla anguilla (European eel) pvalb1
Danio rerio (zebrafish) pvalb1
Onychostoma macrolepis pvalb1
Ictalurus punctatus (channel catfish) pvalb1
Seriola lalandi (yellowtail amberjack) pvalb1
Seriola dumerili (greater amberjack) pvalb1
Sparus aurata (Gilthead seabream) pvalb1
Takifugu rubripes (fugu) pvalb1
Oryzias latipes (Japanese medaka drRD) pvalb1
Scophthalmus maximus (turbot) pvalb1
Oreochromis niloticus (Nile tilapia) pvalb1
Polypterus senegalus (gray bichir) 2990
Polypterus senegalus (gray bichir) 2675
Lepisosteus oculatus (spotted gar) 8560
Lepisosteus oculatus (spotted gar) 8573
Ictalurus punctatus (channel catfish) pvalb3
Danio rerio (zebrafish) pvalb3
Cyprinus carpio (common carp) cyp c 1.01 pvalb3 (Chr.B12)
Onychostoma macrolepis pvalb3
Gadus morhua (Atlantic cod) Gad m 1.0101 pvalb2
Danio rerio (zebrafish) pvalb2
Onychostoma macrolepis pvalb2
Ictalurus punctatus (channel catfish) pvalb2
Plecoglossus altivelis (ayu) pvalb2
Esox lucius (Northern pike) pvalb2B
Gadus morhua (Atlantic cod) pvalb1
Esox lucius (Northern pike) pvalb2A
Clupea harengus (Atlantic herring) pvalb2A
Clupea harengus (Atlantic herring) pvalb2B
Paramormyrops kingsleyae (elephant fish) pvalb1
Paramormyrops kingsleyae (elephant fish) pvalb2
Polypterus senegalus (gray bichir) 3078
Lepisosteus oculatus (spotted gar) 8585
Latimeria chalumnae (coelacanth) XP 005998419
Gallus gallus (chicken) 1286
Xenopus tropicalis (tropical clawed frog) OCM4.1
Xenopus tropicalis (tropical clawed frog) OCM4.6
Xenopus tropicalis (tropical clawed frog) OCM4.5
Xenopus tropicalis (tropical clawed frog) OCM4.4
Xenopus tropicalis (tropical clawed frog) OCM4.2
Xenopus tropicalis (tropical clawed frog) OCM4.3
Polypterus senegalus (gray bichir) 6869
Polypterus senegalus (gray bichir) 6197
Lepisosteus oculatus (spotted gar) 8594
PVALB5
73
Danio rerio (zebrafish) pvalb5
Onychostoma macrolepis pvalb5
Ictalurus punctatus (channel catfish) pvalb5
Anguilla anguilla (European eel) pvalb5
Clupea harengus (Atlantic herring) pvalb5
Salmo salar (Atlantic salmon) pvalb5 (Chr.6)
Seriola lalandi (yellowtail amberjack) pvalb5
Seriola dumerili (greater amberjack) pvalb5
Takifugu rubripes (fugu) pvalb5
Sparus aurata (gilthead seabream) pvalb5
Scophthalmus maximus (turbot) pvalb5
Oreochromis niloticus (Nile tilapia) pvalb5
Oryzias latipes (Japanese medaka drRD) pvalb5
Mustelus griseus (spotless smooth-hound shark) SPV-II
Latimeria chalumnae (coelacanth) XP 005998420
Talpa occidentalis (Iberian mole) XP 037360422
Xenopus tropicalis (tropical clawed frog) OCM3
Pelophylax lessonae (pool frog) pvalb-beta
Amphiuma means (two-toed amphiuma) pvalb-beta
Sarcophilus harrisii (Tasmanian devil) XP 003761513
Gallus gallus (chicken) ATH
Ornithorhynchus anatinus (platypus) XP 001512095
Latimeria chalumnae (coelacanth) XP 005998418
PVALB10
65
Polypterus senegalus (gray bichir) 2892
Lepisosteus oculatus (spotted gar) 8606
Anguilla anguilla (European eel) pvalb10
Clupea harengus (Atlantic herring) pvalb10
Salmo salar (Atlantic salmon) pvalb10 (Chr.28)
Onychostoma macrolepis pvalb10
Gadus morhua (Atlantic cod) pvalb10
Sparus aurata (gilthead seabream) pvalb10
Takifugu rubripes (fugu) pvalb10
Scophthalmus maximus (Turbot) pvalb10
Seriola lalandi (yellowtail amberjack) pvalb10
Seriola dumerili (greater amberjack) pvalb10
PVALB-α
87
Xenopus tropicalis (tropical clawed frog) pvalb-alpha
Pelophylax lessonae (pool frog) pvalb-alpha
Gallus gallus (chicken) pvalb-alpha (CPV1)
Ornithorhynchus anatinus (platypus) pvalb-alpha
Sarcophilus harrisii (Tasmanian devil) pvalb-alpha
Homo sapiens (human) pvalb-alpha
Talpa occidentalis (Iberian mole) pvalb-alpha
Mus musculus (mouse) pvalb-alpha
Callorhinchus milii (elephant shark) pvalb-alpha
Amphiuma means (two-toed amphiuma) pvalb-alpha
Triakis semifasciata (leopard shark) pvalb-alpha
Mustelus griseus (spotless smooth-hound shark) SPV-I
Latimeria chalumnae (coelacanth) pvalb-alpha
Polypterus senegalus (gray bichir) pvalb-alpha
Lepisosteus oculatus (spotted gar) pvalb-alpha
Seriola lalandi (yellowtail amberjack) pvalb7
Seriola dumerili (greater amberjack) pvalb7
Oreochromis niloticus (Nile tilapia) pvalb7
Oryzias latipes (Japanese medaka drRD) pvalb7
Takifugu rubripes (fugu) pvalb7
Sparus aurata (gilthead seabream) pvalb7
Scophthalmus maximus (turbot) pvalb7
Gadus morhua (Atlantic cod) pvalb7
Esox lucius (Northern pike) pvalb7
Clupea harengus (Atlantic herring) pvalb7
Danio rerio (zebrafish)-pvalb7
Onychostoma macrolepis pvalb7
Ictalurus punctatus (channel catfish) pvalb7
Anguilla anguilla (European eel) pvalb6
Paramormyrops kingsleyae (elephant fish) pvalb6
Paramormyrops kingsleyae (elephant fish) pvalb7
Anguilla anguilla (European eel) pvalb7
Danio rerio (zebrafish) pvalb6
Onychostoma macrolepis pvalb6
Ictalurus punctatus (channel catfish) pvalb6
Clupea harengus (Atlantic herring) pvalb6
Gadus morhua (Atlantic cod) pvalb6
Esox lucius (Northern pike) pvalb6
Sparus aurata (gilthead seabream) pvalb6
Scophthalmus maximus (turbot) pvalb6
Seriola lalandi (yellowtail amberjack) pvalb6
Seriola dumerili (greater amberjack) pvalb6
Oryzias latipes (Japanese medaka drRD) pvalb6
Takifugu rubripes (fugu) pvalb6
Oreochromis niloticus (Nile tilapia) pvalb6
Oncomodulin
65
Callorhinchus milii (elephant shark) parvalbumin 6258
Homo sapiens (human) oncomodulin-1
Homo sapiens (human) oncomodulin-2
Talpa occidentalis (Iberian mole) oncomodulin
Mus musculus (mouse) oncomodulin
Sarcophilus harrisii (Tasmanian devil) oncomodulin
Ornithorhynchus anatinus (platypus) oncomodulin
Gallus gallus (chicken) oncomodulin (CPV3)
Latimeria chalumnae (coelacanth) oncomodulin
Xenopus tropicalis (tropical clawed frog) OCM1
Paramormyrops kingsleyae (elephant fish) pvalb8
Polypterus senegalus (gray bichir) 3080
Lepisosteus oculatus (spotted gar) 8552
Ictalurus punctatus (channel catfish) pvalb8
Anguilla anguilla (European eel) pvalb9
Clupea harengus (Atlantic herring) pvalb8
Esox lucius (Northern pike) pvalb8
Danio rerio (zebrafish) pvalb8
Onychostoma macrolepis pvalb8
Scophthalmus maximus (turbot) pvalb8
Sparus aurata (giltead seabream) pvalb8
Seriola lalandi (yellowtail amberjack) pvalb8
Seriola dumerili (greater amberjack) pvalb8
Takifugu rubripes (fugu) pvalb8
Oreochromis niloticus (Nile tilapia) pvalb8
Oryzias latipes (Japanese medaka drRD) pvalb8
Gadus morhua (Atlantic cod) pvalb8
Anguilla anguilla (European eel) pvalb8
Paramormyrops kingsleyae (elephant fish) pvalb9
Clupea harengus (Atlantic herring) pvalb9
Ictalurus punctatus (channel catfish) pvalb9
Scophthalmus maximus (turbot) pvalb9
Oreochromis niloticus (Nile tilapia) pvalb9
Esox lucius (Northern pike) pvalb9
Gadus morhua (Atlantic cod) pvalb9
Danio rerio (zebrafish)-pvalb9
Onychostoma macrolepis pvalb9
Oryzias latipes (Japanese medaka drRD) pvalb9
Takifugu rubripes (fugu) pvalb9
Sparus aurata (giltead seabream) pvalb9
Seriola lalandi (yellowtail amberjack) pvalb9
Seriola dumerili (greater amberjack) pvalb9
Callorhinchus milii (elephant shark) 6255
Callorhinchus milii (elephant shark) 6256
Callorhinchus milii (elephant shark) 1941
Homo sapiens (human) calmodulin-1
Sparus aurata (Gilthead seabream) pvalb-like
Anguilla anguilla (European eel) pvalb-like
0.2

(B) NJ tree

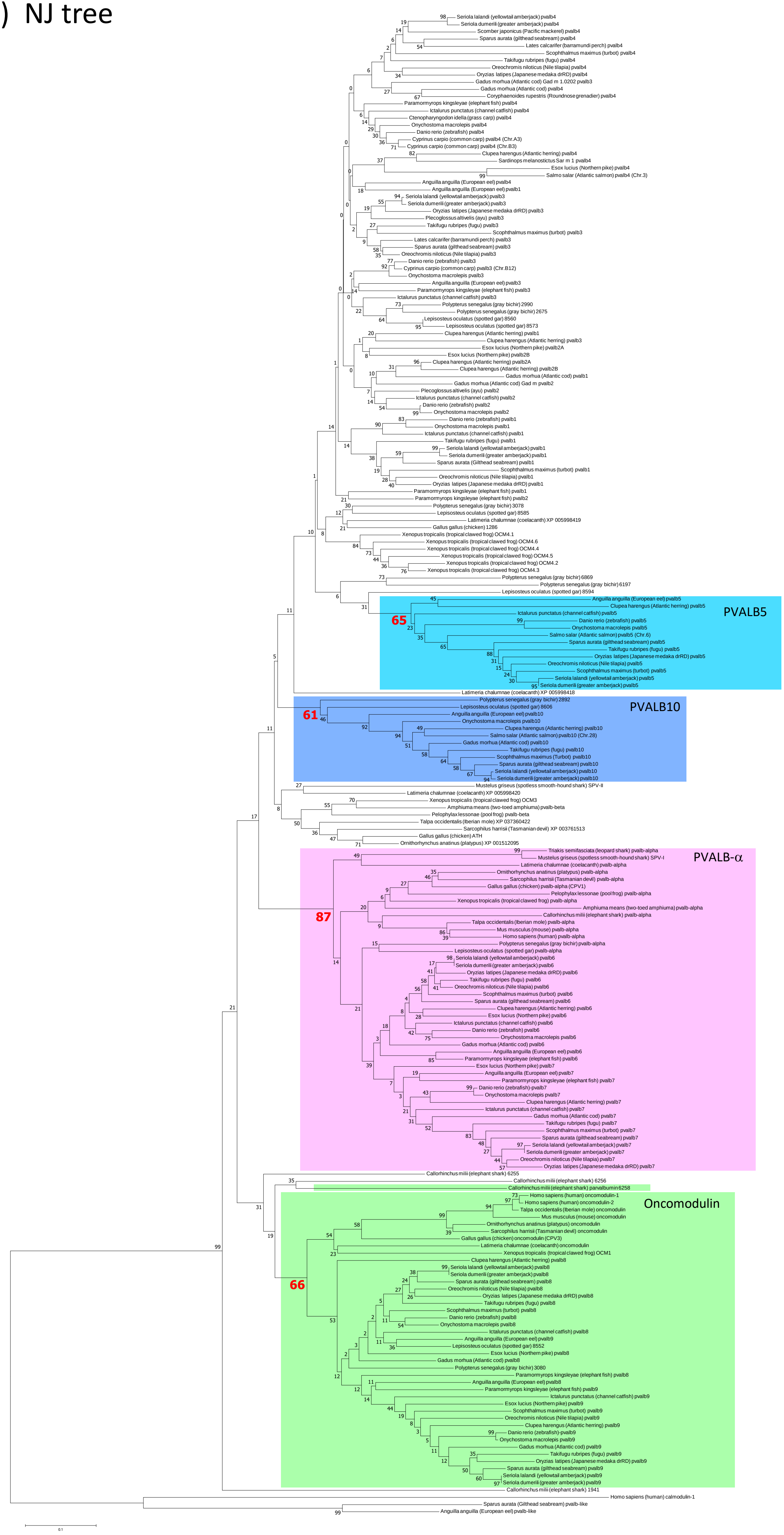

Seriola lalandi (yellowtail amberjack) pvalb4
Seriola dumerili (greater amberjack) pvalb4
Scomber japonicus (Pacific mackerel) pvalb4
Sparus aurata (gilthead seabream) pvalb4
Lates calcarifer (barramundi perch) pvalb4
Scophthalmus maximus (turbot) pvalb4
Takifugu rubripes (fugu) pvalb4
Oreochromis niloticus (Nile tilapia) pvalb4
Oryzias latipes (Japanese medaka drRD) pvalb4
Gadus morhua (Atlantic cod) Gad m 1.0202 pvalb3
Gadus morhua (Atlantic cod) pvalb4
Coryphaenoides rupestris (Roundnose grenadier) pvalb4
Paramormyrops kingsleyae (elephant fish) pvalb4
Ictalurus punctatus (channel catfish) pvalb4
Ctenopharyngodon idella (grass carp) pvalb4
Onychostoma macrolepis pvalb4
Danio rerio (zebrafish) pvalb4
Cyprinus carpio (common carp) pvalb4 (Chr.A3)
Cyprinus carpio (common carp) pvalb4 (Chr.B3)
Clupea harengus (Atlantic herring) pvalb4
Sardinops melanostictus Sar m 1 pvalb4
Esox lucius (Northern pike) pvalb4
Salmo salar (Atlantic salmon) pvalb4 (Chr.3)
Anguilla anguilla (European eel) pvalb4
Anguilla anguilla (European eel) pvalb1
Seriola lalandi (yellowtail amberjack) pvalb3
Seriola dumerili (greater amberjack) pvalb3
Oryzias latipes (Japanese medaka drRD) pvalb3
Plecoglossus altivelis (ayu) pvalb3
Takifugu rubripes (fugu) pvalb3
Scophthalmus maximus (turbot) pvalb3
Lates calcarifer (barramundi perch) pvalb3
Sparus aurata (gilthead seabream) pvalb3
Oreochromis niloticus (Nile tilapia) pvalb3
Danio rerio (zebrafish) pvalb3
Cyprinus carpio (common carp) pvalb3 (Chr.B12)
Onychostoma macrolepis pvalb3
Anguilla anguilla (European eel) pvalb3
Paramormyrops kingsleyae (elephant fish) pvalb3
Ictalurus punctatus (channel catfish) pvalb3
Polypterus senegalus (gray bichir) 2990
Polypterus senegalus (gray bichir) 2675
Lepisosteus oculatus (spotted gar) 8560
Lepisosteus oculatus (spotted gar) 8573
Clupea harengus (Atlantic herring) pvalb1
Clupea harengus (Atlantic herring) pvalb3
Esox lucius (Northern pike) pvalb2A
Esox lucius (Northern pike) pvalb2B
Clupea harengus (Atlantic herring) pvalb2A
Clupea harengus (Atlantic herring) pvalb2B
Gadus morhua (Atlantic cod) pvalb1
Gadus morhua (Atlantic cod) Gad m pvalb2
Plecoglossus altivelis (ayu) pvalb2
Ictalurus punctatus (channel catfish) pvalb2
Danio rerio (zebrafish) pvalb2
Onychostoma macrolepis pvalb2
Danio rerio (zebrafish) pvalb1
Onychostoma macrolepis pvalb1
Ictalurus punctatus (channel catfish) pvalb1
Takifugu rubripes (fugu) pvalb1
Seriola lalandi (yellowtail amberjack) pvalb1
Seriola dumerili (greater amberjack) pvalb1
Sparus aurata (Gilthead seabream) pvalb1
Scophthalmus maximus (turbot) pvalb1
Oreochromis niloticus (Nile tilapia) pvalb1
Oryzias latipes (Japanese medaka drRD) pvalb1
Paramormyrops kingsleyae (elephant fish) pvalb1
Paramormyrops kingsleyae (elephant fish) pvalb2
Polypterus senegalus (gray bichir) 3078
Lepisosteus oculatus (spotted gar) 8585
Latimeria chalumnae (coelacanth) XP 005998419
Gallus gallus (chicken) 1286
Xenopus tropicalis (tropical clawed frog) OCM4.1
Xenopus tropicalis (tropical clawed frog) OCM4.6
Xenopus tropicalis (tropical clawed frog) OCM4.4
Xenopus tropicalis (tropical clawed frog) OCM4.5
Xenopus tropicalis (tropical clawed frog) OCM4.2
Xenopus tropicalis (tropical clawed frog) OCM4.3
Polypterus senegalus (gray bichir) 6869
Polypterus senegalus (gray bichir) 6197
Lepisosteus oculatus (spotted gar) 8594
PVALB5
65
Anguilla anguilla (European eel) pvalb5
Clupea harengus (Atlantic herring) pvalb5
Ictalurus punctatus (channel catfish) pvalb5
Danio rerio (zebrafish) pvalb5
Onychostoma macrolepis pvalb5
Salmo salar (Atlantic salmon) pvalb5 (Chr.6)
Sparus aurata (gilthead seabream) pvalb5
Takifugu rubripes (fugu) pvalb5
Oryzias latipes (Japanese medaka drRD) pvalb5
Oreochromis niloticus (Nile tilapia) pvalb5
Scophthalmus maximus (turbot) pvalb5
Seriola lalandi (yellowtail amberjack) pvalb5
Seriola dumerili (greater amberjack) pvalb5
Latimeria chalumnae (coelacanth) XP 005998418
PVALB10
61
Polypterus senegalus (gray bichir) 2892
Lepisosteus oculatus (spotted gar) 8606
Anguilla anguilla (European eel) pvalb10
Onychostoma macrolepis pvalb10
Clupea harengus (Atlantic herring) pvalb10
Salmo salar (Atlantic salmon) pvalb10 (Chr.28)
Gadus morhua (Atlantic cod) pvalb10
Takifugu rubripes (fugu) pvalb10
Scophthalmus maximus (Turbot) pvalb10
Sparus aurata (gilthead seabream) pvalb10
Seriola lalandi (yellowtail amberjack) pvalb10
Seriola dumerili (greater amberjack) pvalb10
Mustelus griseus (spotless smooth-hound shark) SPV-II
Latimeria chalumnae (coelacanth) XP 005998420
Xenopus tropicalis (tropical clawed frog) OCM3
Amphiuma means (two-toed amphiuma) pvalb-beta
Pelophylax lessonae (pool frog) pvalb-beta
Talpa occidentalis (iberian mole) XP 037360422
Sarcophilus harrisii (Tasmanian devil) XP 003761513
Gallus gallus (chicken) ATH
Ornithorhynchus anatinus (platypus) XP 001512095
PVALB-α
87
Triakis semifasciata (leopard shark) pvalb-alpha
Mustelus griseus (spotless smooth-hound shark) SPV-I
Latimeria chalumnae (coelacanth) pvalb-alpha
Ornithorhynchus anatinus (platypus) pvalb-alpha
Sarcophilus harrisii (Tasmanian devil) pvalb-alpha
Gallus gallus (chicken) pvalb-alpha (CPV1)
Pelophylax lessonae (pool frog) pvalb-alpha
Xenopus tropicalis (tropical clawed frog) pvalb-alpha
Amphiuma means (two-toed amphiuma) pvalb-alpha
Callorhinchus milii (elephant shark) pvalb-alpha
Talpa occidentalis (iberian mole) pvalb-alpha
Mus musculus (mouse) pvalb-alpha
Homo sapiens (human) pvalb-alpha
Polypterus senegalus (gray bichir) pvalb-alpha
Lepisosteus oculatus (spotted gar) pvalb-alpha
Seriola lalandi (yellowtail amberjack) pvalb6
Seriola dumerili (greater amberjack) pvalb6
Oryzias latipes (Japanese medaka drRD) pvalb6
Takifugu rubripes (fugu) pvalb6
Oreochromis niloticus (Nile tilapia) pvalb6
Scophthalmus maximus (turbot) pvalb6
Sparus aurata (gilthead seabream) pvalb6
Clupea harengus (Atlantic herring) pvalb6
Esox lucius (Northern pike) pvalb6
Ictalurus punctatus (channel catfish) pvalb6
Danio rerio (zebrafish) pvalb6
Onychostoma macrolepis pvalb6
Gadus morhua (Atlantic cod) pvalb6
Anguilla anguilla (European eel) pvalb6
Paramormyrops kingsleyae (elephant fish) pvalb6
Esox lucius (Northern pike) pvalb7
Anguilla anguilla (European eel) pvalb7
Paramormyrops kingsleyae (elephant fish) pvalb7
Danio rerio (zebrafish)-pvalb7
Onychostoma macrolepis pvalb7
Clupea harengus (Atlantic herring) pvalb7
Ictalurus punctatus (channel catfish) pvalb7
Gadus morhua (Atlantic cod) pvalb7
Takifugu rubripes (fugu) pvalb7
Scophthalmus maximus (turbot) pvalb7
Sparus aurata (gilthead seabream) pvalb7
Seriola lalandi (yellowtail amberjack) pvalb7
Seriola dumerili (greater amberjack) pvalb7
Oreochromis niloticus (Nile tilapia) pvalb7
Oryzias latipes (Japanese medaka drRD) pvalb7
Callorhinchus milii (elephant shark) 6255
Callorhinchus milii (elephant shark) 6256
Callorhinchus milii (elephant shark) parvalbumin 6258
Oncomodulin
66
Homo sapiens (human) oncomodulin-1
Homo sapiens (human) oncomodulin-2
Talpa occidentalis (iberian mole) oncomodulin
Mus musculus (mouse) oncomodulin
Ornithorhynchus anatinus (platypus) oncomodulin
Sarcophilus harrisii (Tasmanian devil) oncomodulin
Gallus gallus (chicken) oncomodulin (CPV3)
Latimeria chalumnae (coelacanth) oncomodulin
Xenopus tropicalis (tropical clawed frog) OCM1
Clupea harengus (Atlantic herring) pvalb8
Seriola lalandi (yellowtail amberjack) pvalb8
Seriola dumerili (greater amberjack) pvalb8
Sparus aurata (gilthead seabream) pvalb8
Oreochromis niloticus (Nile tilapia) pvalb8
Oryzias latipes (Japanese medaka drRD) pvalb8
Takifugu rubripes (fugu) pvalb8
Scophthalmus maximus (turbot) pvalb8
Danio rerio (zebrafish) pvalb8
Onychostoma macrolepis pvalb8
Ictalurus punctatus (channel catfish) pvalb8
Anguilla anguilla (European eel) pvalb9
Lepisosteus oculatus (spotted gar) 8552
Esox lucius (Northern pike) pvalb8
Gadus morhua (Atlantic cod) pvalb8
Polypterus senegalus (gray bichir) 3080
Paramormyrops kingsleyae (elephant fish) pvalb8
Anguilla anguilla (European eel) pvalb8
Paramormyrops kingsleyae (elephant fish) pvalb9
Ictalurus punctatus (channel catfish) pvalb9
Esox lucius (Northern pike) pvalb9
Scophthalmus maximus (turbot) pvalb9
Oreochromis niloticus (Nile tilapia) pvalb9
Clupea harengus (Atlantic herring) pvalb9
Danio rerio (zebrafish)-pvalb9
Onychostoma macrolepis pvalb9
Gadus morhua (Atlantic cod) pvalb9
Takifugu rubripes (fugu) pvalb9
Oryzias latipes (Japanese medaka drRD) pvalb9
Sparus aurata (gilthead seabream) pvalb9
Seriola lalandi (yellowtail amberjack) pvalb9
Seriola dumerili (greater amberjack) pvalb9
Callorhinchus milii (elephant shark) 1941
Homo sapiens (human) calmodulin-1
Sparus aurata (Gilthead seabream) pvalb-like
Anguilla anguilla (European eel) pvalb-like

(C) Minimum Evolution tree

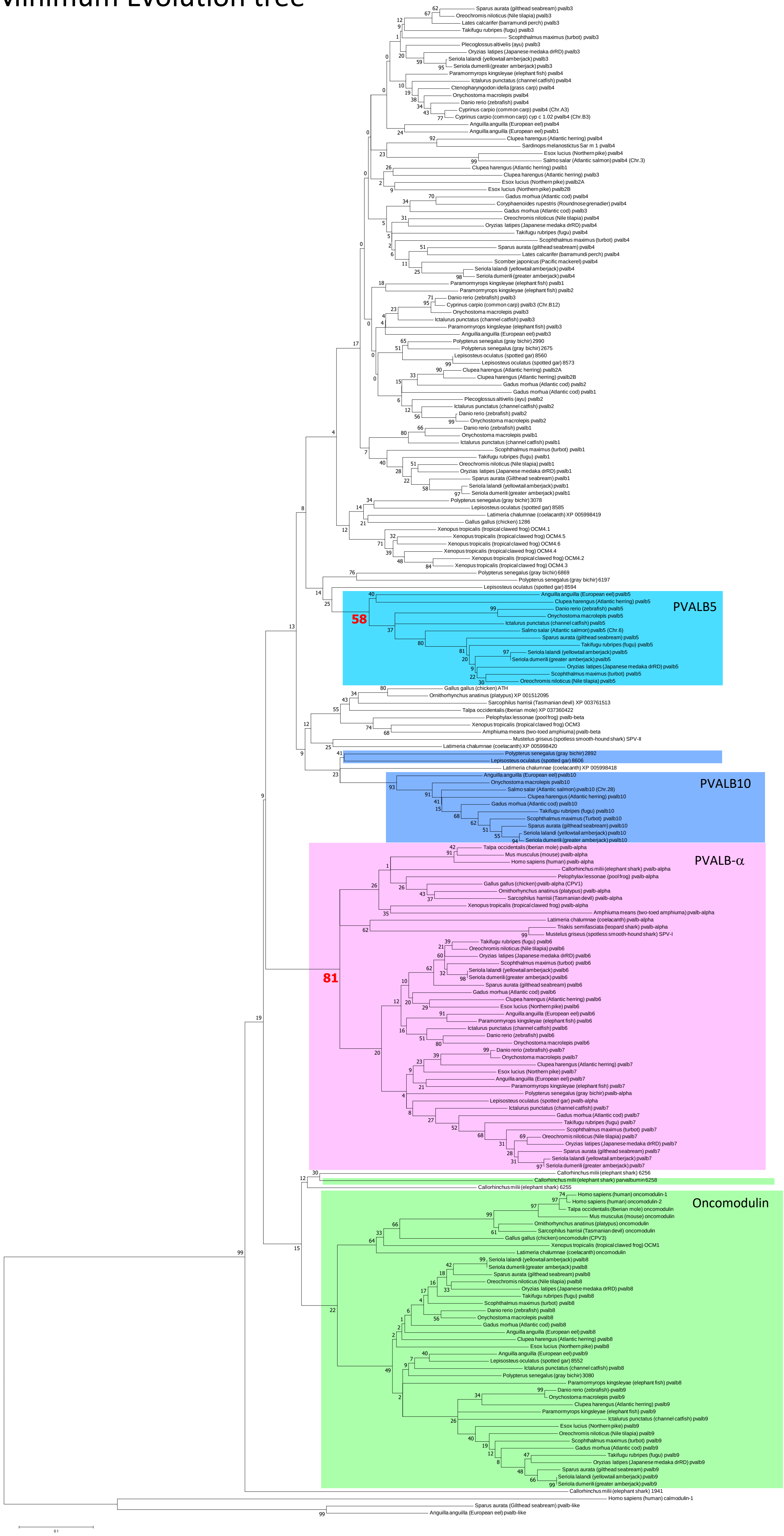

(D) UPGMA tree

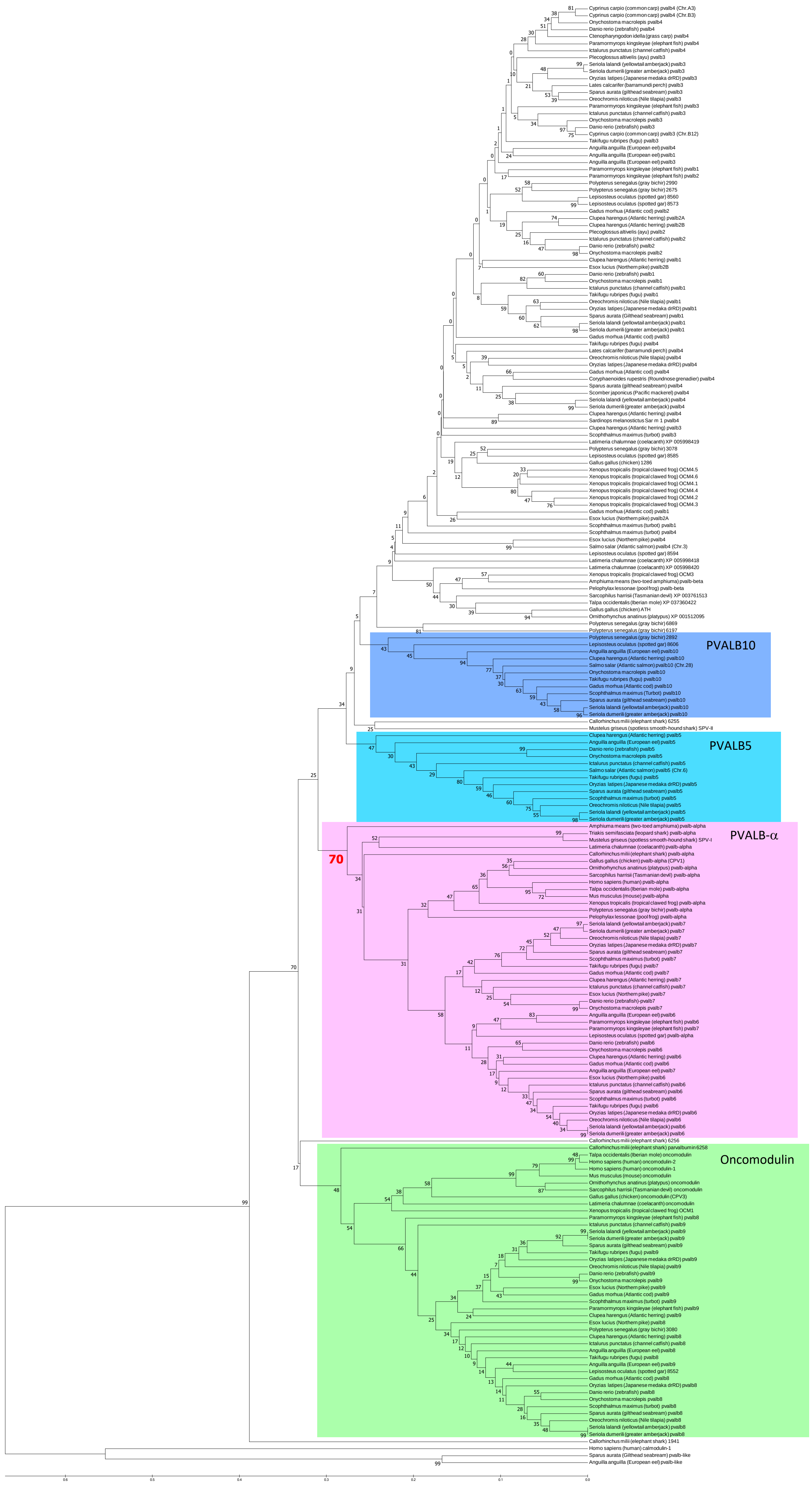

Cyprinus carpio (common carp) pvalb4 (Chr.A3)
Cyprinus carpio (common carp) pvalb4 (Chr.B3)
Onychostoma macrolepis pvalb4
Danio rerio (zebrafish) pvalb4
Ctenopharyngodon idella (grass carp) pvalb4
Paramormyrops kingsleyae (elephant fish) pvalb4
Ictalurus punctatus (channel catfish) pvalb4
Plecoglossus altivelis (ayu) pvalb3
Seriola lalandi (yellowtail amberjack) pvalb3
Seriola dumerili (greater amberjack) pvalb3
Oryzias latipes (Japanese medaka drRD) pvalb3
Lates calcarifer (barramundi perch) pvalb3
Sparus aurata (gilthead seabream) pvalb3
Oreochromis niloticus (Nile tilapia) pvalb3
Paramormyrops kingsleyae (elephant fish) pvalb3
Ictalurus punctatus (channel catfish) pvalb3
Onychostoma macrolepis pvalb3
Danio rerio (zebrafish) pvalb3
Cyprinus carpio (common carp) pvalb3 (Chr.B12)
Takifugu rubripes (fugu) pvalb3
Anguilla anguilla (European eel) pvalb4
Anguilla anguilla (European eel) pvalb1
Anguilla anguilla (European eel) pvalb3
Paramormyrops kingsleyae (elephant fish) pvalb1
Paramormyrops kingsleyae (elephant fish) pvalb2
Polypterus senegalus (gray bichir) 2990
Polypterus senegalus (gray bichir) 2675
Lepisosteus oculatus (spotted gar) 8560
Lepisosteus oculatus (spotted gar) 8573
Gadus morhua (Atlantic cod) pvalb2
Clupea harengus (Atlantic herring) pvalb2A
Clupea harengus (Atlantic herring) pvalb2B
Plecoglossus altivelis (ayu) pvalb2
Ictalurus punctatus (channel catfish) pvalb2
Danio rerio (zebrafish) pvalb2
Onychostoma macrolepis pvalb2
Clupea harengus (Atlantic herring) pvalb1
Esox lucius (Northern pike) pvalb2B
Danio rerio (zebrafish) pvalb1
Onychostoma macrolepis pvalb1
Ictalurus punctatus (channel catfish) pvalb1
Takifugu rubripes (fugu) pvalb1
Oreochromis niloticus (Nile tilapia) pvalb1
Oryzias latipes (Japanese medaka drRD) pvalb1
Sparus aurata (Gilthead seabream) pvalb1
Seriola lalandi (yellowtail amberjack) pvalb1
Seriola dumerili (greater amberjack) pvalb1
Gadus morhua (Atlantic cod) pvalb3
Takifugu rubripes (fugu) pvalb4
Lates calcarifer (barramundi perch) pvalb4
Oreochromis niloticus (Nile tilapia) pvalb4
Oryzias latipes (Japanese medaka drRD) pvalb4
Gadus morhua (Atlantic cod) pvalb4
Coryphaenoides rupestris (Roundnose grenadier) pvalb4
Sparus aurata (gilthead seabream) pvalb4
Scomber japonicus (Pacific mackerel) pvalb4
Seriola lalandi (yellowtail amberjack) pvalb4
Seriola dumerili (greater amberjack) pvalb4
Clupea harengus (Atlantic herring) pvalb4
Sardinops melanostictus Sar m 1 pvalb4
Clupea harengus (Atlantic herring) pvalb3
Scophthalmus maximus (turbot) pvalb3
Latimeria chalumnae (coelacanth) XP 005998419
Polypterus senegalus (gray bichir) 3078
Lepisosteus oculatus (spotted gar) 8585
Gallus gallus (chicken) 1286
Xenopus tropicalis (tropical clawed frog) OCM4.5
Xenopus tropicalis (tropical clawed frog) OCM4.6
Xenopus tropicalis (tropical clawed frog) OCM4.1
Xenopus tropicalis (tropical clawed frog) OCM4.4
Xenopus tropicalis (tropical clawed frog) OCM4.2
Xenopus tropicalis (tropical clawed frog) OCM4.3
Gadus morhua (Atlantic cod) pvalb1
Esox lucius (Northern pike) pvalb2A
Scophthalmus maximus (turbot) pvalb1
Scophthalmus maximus (turbot) pvalb4
Esox lucius (Northern pike) pvalb4
Salmo salar (Atlantic salmon) pvalb4 (Chr.3)
Lepisosteus oculatus (spotted gar) 8594
Latimeria chalumnae (coelacanth) XP 005998418
Latimeria chalumnae (coelacanth) XP 005998420
Xenopus tropicalis (tropical clawed frog) OCM3
Amphiuma means (two-toed amphiuma) pvalb-beta
Pelophylax lessonae (pool frog) pvalb-beta
Sarcophilus harrisii (Tasmanian devil) XP 003761513
Talpa occidentalis (Iberian mole) XP 037360422
Gallus gallus (chicken) ATH
Ornithorhynchus anatinus (platypus) XP 001512095
Polypterus senegalus (gray bichir) 6869
Polypterus senegalus (gray bichir) 6197
Polypterus senegalus (gray bichir) 2892
Lepisosteus oculatus (spotted gar) 8606
Anguilla anguilla (European eel) pvalb10
Clupea harengus (Atlantic herring) pvalb10
Salmo salar (Atlantic salmon) pvalb10 (Chr.28)
Onychostoma macrolepis pvalb10
Takifugu rubripes (fugu) pvalb10
Gadus morhua (Atlantic cod) pvalb10
Scophthalmus maximus (Turbot) pvalb10
Sparus aurata (gilthead seabream) pvalb10
Seriola lalandi (yellowtail amberjack) pvalb10
Seriola dumerili (greater amberjack) pvalb10
PVALB10
Callorhinchus milii (elephant shark) 6255
Mustelus griseus (spotless smooth-hound shark) SPV-II
Clupea harengus (Atlantic herring) pvalb5
Anguilla anguilla (European eel) pvalb5
Danio rerio (zebrafish) pvalb5
Onychostoma macrolepis pvalb5
Ictalurus punctatus (channel catfish) pvalb5
Salmo salar (Atlantic salmon) pvalb5 (Chr.6)
Takifugu rubripes (fugu) pvalb5
Oryzias latipes (Japanese medaka drRD) pvalb5
Sparus aurata (gilthead seabream) pvalb5
Scophthalmus maximus (turbot) pvalb5
Oreochromis niloticus (Nile tilapia) pvalb5
Seriola lalandi (yellowtail amberjack) pvalb5
Seriola dumerili (greater amberjack) pvalb5
PVALB5
Amphiuma means (two-toed amphiuma) pvalb-alpha
Triakis semifasciata (leopard shark) pvalb-alpha
Mustelus griseus (spotless smooth-hound shark) SPV-I
Latimeria chalumnae (coelacanth) pvalb-alpha
Callorhinchus milii (elephant shark) pvalb-alpha
Gallus gallus (chicken) pvalb-alpha (CPV1)
Ornithorhynchus anatinus (platypus) pvalb-alpha
Sarcophilus harrisii (Tasmanian devil) pvalb-alpha
Homo sapiens (human) pvalb-alpha
Talpa occidentalis (Iberian mole) pvalb-alpha
Mus musculus (mouse) pvalb-alpha
Xenopus tropicalis (tropical clawed frog) pvalb-alpha
Polypterus senegalus (gray bichir) pvalb-alpha
Pelophylax lessonae (pool frog) pvalb-alpha
Seriola lalandi (yellowtail amberjack) pvalb7
Seriola dumerili (greater amberjack) pvalb7
Oreochromis niloticus (Nile tilapia) pvalb7
Oryzias latipes (Japanese medaka drRD) pvalb7
Sparus aurata (gilthead seabream) pvalb7
Scophthalmus maximus (turbot) pvalb7
Takifugu rubripes (fugu) pvalb7
Gadus morhua (Atlantic cod) pvalb7
Clupea harengus (Atlantic herring) pvalb7
Ictalurus punctatus (channel catfish) pvalb7
Esox lucius (Northern pike) pvalb7
Danio rerio (zebrafish)-pvalb7
Onychostoma macrolepis pvalb7
Anguilla anguilla (European eel) pvalb6
Paramormyrops kingsleyae (elephant fish) pvalb6
Paramormyrops kingsleyae (elephant fish) pvalb7
Lepisosteus oculatus (spotted gar) pvalb-alpha
Danio rerio (zebrafish) pvalb6
Onychostoma macrolepis pvalb6
Clupea harengus (Atlantic herring) pvalb6
Gadus morhua (Atlantic cod) pvalb6
Anguilla anguilla (European eel) pvalb7
Esox lucius (Northern pike) pvalb6
Ictalurus punctatus (channel catfish) pvalb6
Sparus aurata (gilthead seabream) pvalb6
Scophthalmus maximus (turbot) pvalb6
Takifugu rubripes (fugu) pvalb6
Oryzias latipes (Japanese medaka drRD) pvalb6
Oreochromis niloticus (Nile tilapia) pvalb6
Seriola lalandi (yellowtail amberjack) pvalb6
Seriola dumerili (greater amberjack) pvalb6
PVALB-α
70
Callorhinchus milii (elephant shark) 6256
Callorhinchus milii (elephant shark) parvalbumin 6258
Talpa occidentalis (Iberian mole) oncomodulin
Homo sapiens (human) oncomodulin-2
Homo sapiens (human) oncomodulin-1
Mus musculus (mouse) oncomodulin
Ornithorhynchus anatinus (platypus) oncomodulin
Sarcophilus harrisii (Tasmanian devil) oncomodulin
Gallus gallus (chicken) oncomodulin (CPV3)
Latimeria chalumnae (coelacanth) oncomodulin
Xenopus tropicalis (tropical clawed frog) OCM1
Paramormyrops kingsleyae (elephant fish) pvalb8
Ictalurus punctatus (channel catfish) pvalb9
Seriola lalandi (yellowtail amberjack) pvalb9
Seriola dumerili (greater amberjack) pvalb9
Sparus aurata (gilthead seabream) pvalb9
Takifugu rubripes (fugu) pvalb9
Oryzias latipes (Japanese medaka drRD) pvalb9
Oreochromis niloticus (Nile tilapia) pvalb9
Danio rerio (zebrafish)-pvalb9
Onychostoma macrolepis pvalb9
Esox lucius (Northern pike) pvalb9
Gadus morhua (Atlantic cod) pvalb9
Scophthalmus maximus (turbot) pvalb9
Paramormyrops kingsleyae (elephant fish) pvalb9
Clupea harengus (Atlantic herring) pvalb9
Esox lucius (Northern pike) pvalb8
Polypterus senegalus (gray bichir) 3080
Clupea harengus (Atlantic herring) pvalb8
Ictalurus punctatus (channel catfish) pvalb8
Anguilla anguilla (European eel) pvalb8
Takifugu rubripes (fugu) pvalb8
Anguilla anguilla (European eel) pvalb9
Lepisosteus oculatus (spotted gar) 8552
Gadus morhua (Atlantic cod) pvalb8
Oryzias latipes (Japanese medaka drRD) pvalb8
Danio rerio (zebrafish) pvalb8
Onychostoma macrolepis pvalb8
Scophthalmus maximus (turbot) pvalb8
Sparus aurata (gilthead seabream) pvalb8
Oreochromis niloticus (Nile tilapia) pvalb8
Seriola lalandi (yellowtail amberjack) pvalb8
Seriola dumerili (greater amberjack) pvalb8
Oncomodulin
Callorhinchus milii (elephant shark) 1941
Homo sapiens (human) calmodulin-1
Sparus aurata (Gilthead seabream) pvalb-like
Anguilla anguilla (European eel) pvalb-like
